# A Framework for the Multi-Level Fusion of Electronic Nose and Electronic Tongue for Tea Quality Assessment

**DOI:** 10.3390/s17051007

**Published:** 2017-05-03

**Authors:** Ruicong Zhi, Lei Zhao, Dezheng Zhang

**Affiliations:** 1 School of Computer and Communication Engineering, University of Science and Technology Beijing, Beijing 100083, China; zdzchina@126.com; 2 Beijing Key Laboratory of Knowledge Engineering for Materials Science, Beijing 100083, China; 3 China National Institute of Standardization, Beijing 100191, China; zhaolei@cnis.gov.cn

**Keywords:** multi-level fusion, feature fusion, decision fusion, electronic nose, electronic tongue, tea quality assessment

## Abstract

Electronic nose (E-nose) and electronic tongue (E-tongue) can mimic the sensory perception of human smell and taste, and they are widely applied in tea quality evaluation by utilizing the fingerprints of response signals representing the overall information of tea samples. The intrinsic part of human perception is the fusion of sensors, as more information is provided comparing to the information from a single sensory organ. In this study, a framework for a multi-level fusion strategy of electronic nose and electronic tongue was proposed to enhance the tea quality prediction accuracies, by simultaneously modeling feature fusion and decision fusion. The procedure included feature-level fusion (fuse the time-domain based feature and frequency-domain based feature) and decision-level fusion (D-S evidence to combine the classification results from multiple classifiers). The experiments were conducted on tea samples collected from various tea providers with four grades. The large quantity made the quality assessment task very difficult, and the experimental results showed much better classification ability for the multi-level fusion system. The proposed algorithm could better represent the overall characteristics of tea samples for both odor and taste.

## 1. Introduction

Tea quality assessment is crucial for both producers and consumers, and it is a very challenging task due to the presence of innumerable compounds and their diverse contribution to tea quality. The evaluation of tea quality is usually carried out through human sensory analysis, which can provide direct and integrated measurements of various attributes. The gradation of tea is yielded on the basis of experienced tea tasters’ scores, and tea tasters assign the perceived intensities of appearance, aroma, and taste captured by their sense organs [1]. However, human sensory evaluation is subjective, as even the same person may provide different evaluation information in different experiments, and sensory panel are inconsistent due to the individual variability. Moreover, there are various affective factors (e.g., physical or mental states) which make the human sensory evaluation inaccurate [2].

With the increased expectation of high quality and a large quantity of the product, there are many requirements for objective measurements in a fast, accurate, and cost-effective manner [3]. Conventional flavor analysis techniques are high cost and not suitable for online quality control, such as gas chromatography (GC), high-performance liquid chromatography (HPLC), plasma atomic emission spectrometry, and capillary electrophoresis [4].

Electronic intelligent systems (e.g., E-nose and E-tongue) have received considerable attention during the last two decades, since numerous applications have increased in the food industry [3,5]. The E-nose and E-tongue can mimic the sensory perception of human smell and taste, and they are widely applied in tea quality evaluation by utilizing fingerprints of response signals representing the overall information of tea samples. E-tongue can detect the taste of samples, whereas e-nose can identify the odor. Both of them are based on the principle of identifying odor/taste by extracting an overall signature from the comprehensive mixture of the compounds. Usually, an electronic intelligent system consists of a sensor array for chemical detection, along with advanced pattern recognition systems so that the sensor signal data can be automatically and reliably processed.

E-noses and E-tongues have been widely used in many food quality assessment applications, such as fruits (grapes, pears, apples, mangos, pineapples, and peaches), beverages (juice, coffee, tea, wine, and milk), meat, fish, and rice, etc. [3,5,6,7]. There are a great number of studies focusing on tea quality assessment by electronic intelligent instruments. Both E-nose and E-tongue are successfully applied to provide fast and reliable results for tea grade identification, storage time and fermentation processes, where the odor of tea or the taste of tea is considered as the sole attribute [8,9,10,11,12,13,14,15,16,17,18,19].

The intrinsic part of human perception is the fusion of sensors, as multiple sensory organs provide more information which helps to make a better decision than a single sensory pipeline. The electronic intelligent system is carried out in a similar way as human perception, with diverse sensors generating different signature phenomena to fully exploit the characteristics of test samples. Data fusion aims to combine information acquired from multiple sources through different strategies to achieve a better description and to enhance the probability of an accurate classification. Usually, fusion can be categorized into data fusion (low-level fusion), feature fusion (intermediate-level fusion), and decision fusion (high-level fusion). The data fusion strategy employs raw data level fusion by combining the raw sensor response signals into a single signal. Feature fusion concatenates the features extracted from the signal values of the sensors through a variety of feature extraction and selection methods. Decision fusion presents the output of multiple classifiers to achieve a final prediction, and each classifier is trained for each signal source. The decision level fusion combines decisions from multiple sensory pipelines, and this is similar to the fusion mechanism in the human brain [5].

A number of researches reported that a system combining an E-nose and E-tongue improved the performance of an individual system for quality assessment [20,21,22,23,24,25,26,27,28,29]. Most of the researches focused on data fusion and feature fusion, by combining E-nose and E-tongue raw sensor signals or features extracted from raw signal data, and a few researches reported on the decision fusion of multiple electronic intelligent instruments [30,31,32,33].

**Figure 1:**
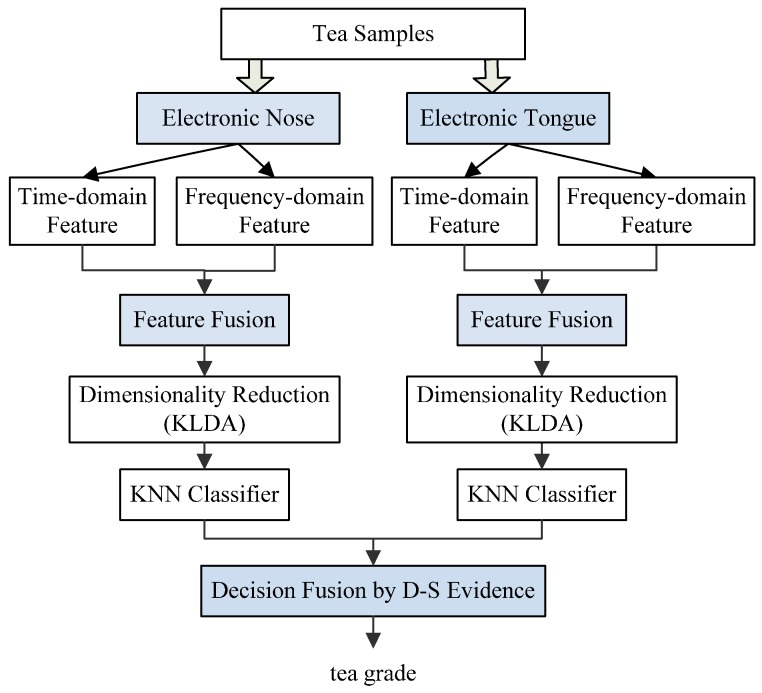
Illustration of the strategy flow of multi-level fusion tea quality assessment.

In this study, a framework for a multi-level fusion strategy of E-nose and E-tongue was proposed to promote the tea quality prediction accuracies by simultaneously modeling feature fusion and decision fusion, and the procedure is illustrated in Figure 1. Two different features of time-domain based and frequency-domain based representations were extracted from the E-nose and E-tongue sensor responses to better represent individual sensor signal characteristics. The merged features were analyzed by a nonlinear dimensionality reduction algorithm for feature selection. Finally, the classification results obtained by the K-nearest neighbor classifier from the E-nose and E-tongue were fused by D-S evidence, which was an effective decision fusion algorithm.

## 2. Materials and Methods

### 2.1. Sample Collection

All of the tea samples belong to a category of green tea, which is called “Longjing tea”, with four different grade levels (grade 1/T, grade 2/Y, grade 3/E, and grade 4/S) selected from seven tea producers (which are denoted by MC, MG, ML, MS, MX, MY, and QD) in the Xihu producing area, Hangzhou, China. The quality of the tea samples was evaluated by national certified tea experts according to the national standard “GB/T 23776-2009 Methodology of sensory evaluation of tea”. Thirty-five tea samples of each grade and each company were used, and there were 980 samples in total. The tea samples were individually vacuum packed with aluminum foil materials, and stored in the refrigerator at −4 °C [34].

### 2.2. Electronic Nose Measurement

An E-nose (Fox 4000, Alpha M.O.S. Co., Toulouse, France) was utilized to acquire the odor fingerprints of the tea samples. The sensor array consists of 18 metallic oxide sensors. The tea sample preparation procedure is as follows: In the preprocessing stage, each tea sample was weighted for 1 g, and mixed with 5 mL ultrapure water at room temperature in a 20 mL headspace bottle. The headspace bottle was arranged in an automatic sampling device after sealing. Cross testing was conducted for different grades of tea samples through a cyclic cross sequence, i.e., all four grades of tea samples were tested in the order of “T, Y, E, S; T, Y, E, S; T, Y, E, S; …”. Therefore, the tea samples with various qualities could be tested alternately, thus avoiding the adaptability of sensors to certain type of tea samples. In the experiments, the headspace bottle was first sent to the preheating area for 900 s, with an oscillator rotation rate of 500 rpm/min and temperature of 60 °C. Then, 2 mL gas was injected into the sensory array room with a speed of 2 mL/s. Before the tea sample detection, pure nitrogen was pumped into the sensor array chamber with a speed of 2 mL/s for 300 s, to ensure that the former gas molecule was thoroughly cleaned out [35,36]. The tea sample reaction time was 120 s, and the responses of the sensors were recorded every 0.5 s. Therefore, there were 241 values for each sensor of the electronic nose.

### 2.3. Electronic Tongue Measurement

An E-tongue (𝛼-ASTREE, Alpha M.O.S. Co., Toulouse, France) with an array of seven electrodes was utilized in this study. The tea samples were prepared by boiling 150 mL of ultrapure water with every 1 g tea and covered. Then, the beaker was put in a boiling water bath (100 °C) and shaken every 10 min. After 45 min, the tea infusion was filtered and cooled down for measurement after 2 h [2]. The electronic tongue is not as stable as the electronic nose, and some preprocessing is needed to enhance the stability and reliability of the tea sample measurement. Such preprocessing includes self-testing, activation, training, calibration, and diagnostics. The procedure is as follows: The connection of the hardware is automatically tested by the equipment software. The sensors are cleaned by deionized water for 10–20 s, and activated in another cup of deionized water for 30 min to enrich H^+^ on the coating film of the sensor. Then, the sensors are trained using HCl (0.01 mol/L, training solution) and ultrapure water (cleaning solution). First, the sensors are cleaned in ultrapure water for 10 s, and then the sensors are moved to the HCl solution for 300 s. The same procedure is repeated for three times which is controlled by the software. Calibration is consequently conducted to check whether the coating film of the sensor is balanced after training. The sensors are cleaned in ultrapure water (10 s) and reacted in 0.01 mol/L HCl (120 s), and the operation is repeated three times. Moreover, the instrument is diagnosed every 20 days in our test to guarantee the sensitivity and effectiveness of the sensors. The procedure is controlled and implemented by the software. Each of the electrodes produced a signal curve, and each signal consisted of 241 points as the signal value was acquired every 0.5 s in the period of 120 s. Therefore, the data formed a matrix of 7 × 241.

### 2.4. Multi-Level Fusion System

#### 2.4.1. Feature Extraction and Fusion

The response signals collected from the E-nose and E-tongue are sequence signals changing over time. It is not feasible to deal with the original signal directly as it is time consuming and there is usually a noisy signal mixed in with the sensor signal sequences. Therefore, feature extraction is of great importance as it can efficiently analyze the sensor sequences and extract the features representing the characteristics of the signals.

Most researches employed time-domain based features, which could represent the inner characteristics from the intelligent sensors’ response signals. In the signal processing field, more effective representations are utilized to denote the frequency-domain based features by filter processing, as they can differentiate the signals which are not well discriminated in the time domain. Therefore, both time-domain based representations and frequency-domain based representations are extracted from the E-nose and E-tongue response signals, and feature fusion is conducted to comprehensively adopt the superiority of both representations.

In this study, the time-domain based representations include the maximum value (MV) and the average value (AV) of the original sensor responses of the E-nose and E-tongue, respectively. The maximum value of the i-th sensor is defined as:

(1) $$MV_{i}=\max\left|x_{i,1},x_{i,2},\ldots ,x_{i,241}\right|\;\left(\begin{array}{l} i=1,2,\ldots ,18\;\mathrm{for}\text{ E-nose}\\ i=1,2,\ldots ,7\;\mathrm{for}\text{ E-tongue} \end{array}\right)$$

The average value of the i-th sensor is defined as:

(2) $$AV_{i}=\frac{x_{i,1}+x_{i,2}+\ldots +x_{i,241}}{241}\;\left(\begin{array}{l} i=1,2,\ldots ,18\;\mathrm{for}\text{ E-nose}\\ i=1,2,\ldots ,7\;\mathrm{for}\text{ E-tongue} \end{array}\right)$$

where i is the sensor label and $x_{i,1},x_{i,2},\ldots ,x_{i,241}$ are the absolute signal of the i-th sensor for the E-nose and E-tongue, respectively.

The frequency-domain based representation is extracted by wavelet packet analysis, and the maximum energy (ME) and average energy (AE) of the wavelet packet coefficients are explored to denote the mainstream traits and the overall level of the sensor signals. The maximum energy (ME) of the i-th sensor is defined as:

(3) $$ME_{i}=\max\left|E_{30}^{i},E_{31}^{i},\ldots ,E_{37}^{i}\right|\;\left(\begin{array}{l} i=1,2,\ldots ,18\;\mathrm{for}\text{ E-nose}\\ i=1,2,\ldots ,7\;\mathrm{for}\text{ E-tongue} \end{array}\right)$$

The average energy (AE) of the i-th sensor is defined as:

(4) $$AE_{i}=\frac{E_{30}^{i},E_{31}^{i},\ldots ,E_{37}^{i}}{8}\;\left(\begin{array}{l} i=1,2,\ldots ,18\;\mathrm{for}\text{ E-nose}\\ i=1,2,\ldots ,7\;\mathrm{for}\text{ E-tongue} \end{array}\right)$$

where $E_{30}^{i},E_{31}^{i},\ldots ,E_{37}^{i}$ are the energies of the *i*-th sensor at each frequency band, and $E_{3j}=\sum_{k=1}^{m}{\left|C_{3jk}\right|}^{2}\left(j=0,1,\ldots ,7\right)$. m is the number of coefficients and $C_{3jk}$ is the k-th coefficient of the j-th frequency band corresponding to three scale orthogonal wavelet decomposition.

The feature-level fusion is conducted by connecting the time-domain based features and frequency-based features in series, i.e., the four different features of each sensor are individually arrayed as ($MV_{i},\;AV_{i},\;ME_{i},\;AE_{i}$), and the fused features of each sensor are concatenated in order of the E-nose and E-tongue, respectively [36]. The fused feature is standardized for further analysis.

#### 2.4.2. Nonlinear Subspace Embedding

Linear separation in original data space is rare in the practical application of quality classification due to the correlation and redundancy among multiple electronic sensors. Therefore, the fused features concatenating time-domain representation and frequency-domain representation extracted from the original sensor signals are embedded in a high dimensional subspace which is linearly separable for the samples. Kernel-based algorithms widely concern nonlinear embedding methods for dimensionality reduction. The effectiveness of kernel-based dimensionality reduction methods was evaluated in [19]. KLDA (Kernel Linear Discriminant Analysis) is utilized in this study to reduce the fused feature dimension [37].

The fused features for sample k (k=1,2,…N) are denoted by $x_{k}=[MV_{i},\;AV_{i},\;ME_{i},\;AE_{i}]$ for the E-nose (i=1,2,…,18) and E-tongue (i=1,2,…,7). In the following, we use a single symbol $X=\left[x_{1};x_{2};\ldots ;x_{N}\right]$ to represent the E-nose and E-tongue data matrix for simplicity. The data matrix X is implicitly mapped to a high dimensional feature space F with a nonlinear transformation Φ, i.e., $F=\left\{\Phi \left(X\right):X\in R^{N}\right\}$, where N is the sample number. The objective of the KLDA algorithm is to maximize the ratio of the between-class scatter and the within-class scatter in F, where the within-class scatter is defined as $S_{w}^{\Phi }=\sum_{i=1}^{c}\sum_{j=1}^{n_{i}}\left(\Phi \left(x_{ij}\right)-m_{i}\right){\left(\Phi \left(x_{ij}\right)-m_{i}\right)}^{T}$, where c is the number of classes (four grades), $n_{i}$ is the number of samples in class i, and $m_{i}$ is the mean of the i-th class sample. The between-class scatter is defined as $S_{b}^{\Phi }=\sum_{i=1}^{c}n_{i}\left(m_{i}-m\right){\left(m_{i}-m\right)}^{T}$, where m is the mean of all the tea samples.

#### 2.4.3. Classification

Classification is the grade identification process on the basis of fused and selected features. The *K*-nearest neighbors (KNN) classifier is a non-parametric method which is among the simplest of the machine learning algorithms employed for classification. The output of the classifier is the class label for the new unclassified sample by a majority vote of its K-nearest neighbors in the training set, and no explicit training step is required.

#### 2.4.4. Decision Fusion Based on D-S Evidence

D-S evidence has been widely applied to artificial intelligence and multi-sensor fusion [38]. The D-S evidence theory is a more general and flexible method than traditional probability approaches, as it supports both the imprecision and uncertainty representation [39]. The basic principle of the D-S evidence is stated in detail, as follows:

Let $\Theta =\left\{\theta_{1},\theta_{2},\ldots ,\theta_{c}\right\}$ be the set of $\theta_{c}\left(\theta_{c}\in \Theta \right)$ corresponding to c identifiable classes, then Θ is the space of the hypotheses called a frame of discernment.

A key issue of the D-S evidence theory is the definition of basic probability assignment. The basic probability assignment function m is defined on $2^{\Theta }$ as $m:2^{\Theta }\to \left[0,1\right]$ for every element A of $2^{\Theta }$. The probability assignment value m(A) satisfies the following properties:

(5) $$\begin{array}{l} m\left(\phi \right)=0\\ \sum_{A\in 2^{\Theta }}m\left(A\right)=1 \end{array}$$

For any $A\in 2^{\Theta }$, the quantity m(A) represents a measure of belief that is exactly committed to A. ϕ is the empty set. The element A of $2^{\Theta }$ with m(A) being greater than zero is called the focal element of m.

The other key issue of the D-S evidence theory is the aggregate multiple evidence from different sources defined on the same frame of discernment by means of the basic probability assignment function.

Two bodies of evidence, $m_{1}$ and $m_{2}$, with focal elements $A_{1},A_{2},\ldots ,A_{i}$ and $B_{1},B_{2},\ldots ,B_{j}$, respectively, can be merged to obtain a new basic probability assignment function m by a combination rule. The D-S evidence combination rule is defined as:

(6) $$m\left(A\right)=\frac{\sum_{A_{i}\cap B_{j}}m_{1}\left(A_{i}\right)m_{2}\left(B_{j}\right)}{1-Q}\;A\neq \phi$$

where $Q=\sum_{A_{i}\cap B_{j}=\phi }m_{1}\left(A_{i}\right)m_{2}\left(B_{j}\right)$ and Q<1.

One of the main difficulties in the D-S evidence is how to initialize the basic probability assignment function as well as possible. There is no general answer to the key problem of the basic probability assignment function definition [40,41]. Generally, the probability values are determined artificially depending on the specific applications.

#### 2.4.5. Decision Fusion for the E-Nose and E-Tongue

The grade identification is conducted by D-S evidence decision fusion for the E-nose and E-tongue, and the KNN classifier is utilized for the E-nose and E-tongue, respectively.

Let the four grades of the tea samples be denoted by T, Y, E, and S. Therefore, the identifiable set is defined as Θ={T,Y,E,S}. In our experiment, the basic probability assignment function is defined by the output of the classifier. The results of the KNN classifier are utilized to calculate the membership degree from the *K*-nearest neighbors of each class, that is:

(7) $$m\left(i\right)=\frac{x\_neigh_{i}}{K},\;i=T,Y,E,S$$

where $x\_neigh_{i}$ is the number of neighbors belonging to class i from the K-nearest neighbors. The parameter K is determined according to the recognition accuracies of the experiments.

Assume that $m_{T}^{i},\;m_{Y}^{i},\;m_{E}^{i},\;m_{S}^{i}$ and $n_{T}^{i},\;n_{Y}^{i},\;n_{E}^{i},\;n_{S}^{i}$ are the basic probability assignment values for the four grades of the i-th tea samples by the E-nose and E-tongue, respectively. The final probability values of the tea sample $M_{T}^{i},\;M_{Y}^{i},\;M_{E}^{i},\;M_{S}^{i}$ are identified by the D-S evidence combination rule, expressed as Equation (6).

The grade of a test tea sample is classified according to the maximum membership rule by the final probability values, that is:

(8) $$class\left(i\right)=suffix\left(\max\left\{M_{j}^{i}\right\}\right),\;j=T,Y,E,S$$

The suffix of the maximum value of $M_{T}^{i},\;M_{Y}^{i},\;M_{E}^{i},\;M_{S}^{i}$ is the grade that the test sample is assigned. The algorithms are implemented by Matlab R2015b (Mathworks, Natick, MA, USA).

## 3. Results and Discussion

### 3.1. Feature Representation from Sensors

Both the time-domain based features (MV, AV) and the frequency-domain based features (ME, AE) were calculated from the sequence signal of the E-nose and E-tongue to enhance the discriminative ability of the features. The feature fusion was individually conducted for the E-nose and E-tongue. The fused features of all the 18 sensors of the E-nose were concatenated in order, and obtained a feature vector of 72 dimensions. Similarly, a vector of 28 dimensions was obtained by fusing the features of all the seven sensors of the electronic tongue.

**Figure 2:**
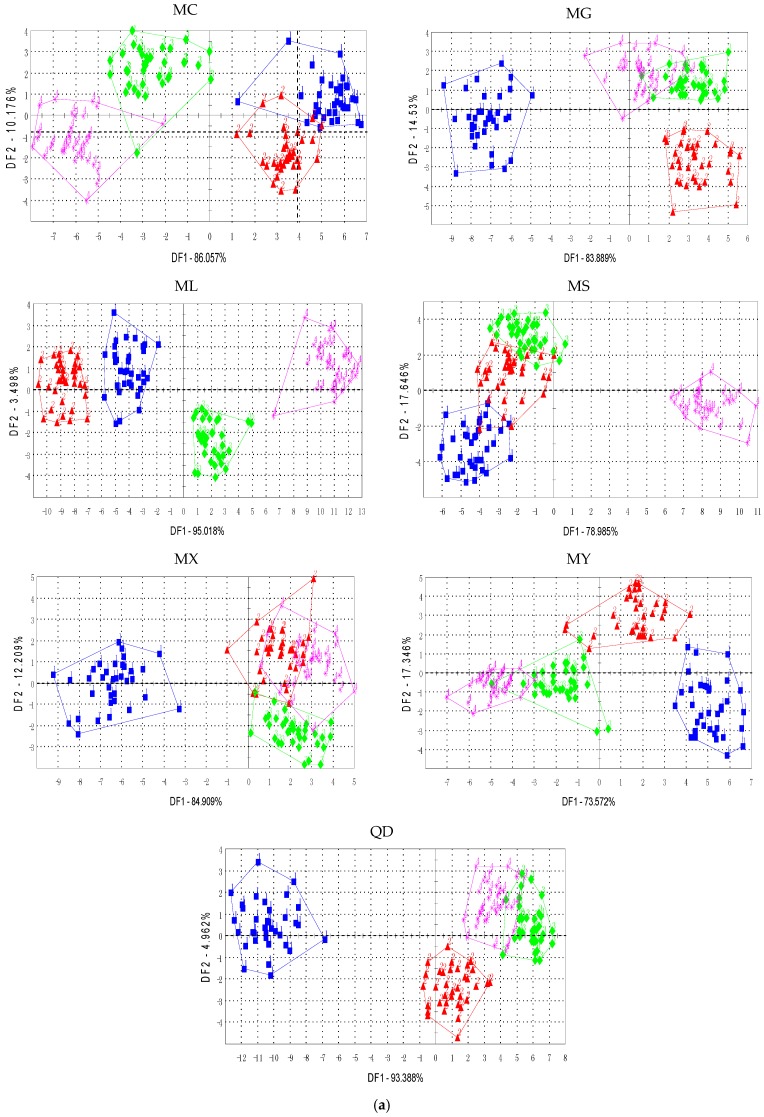

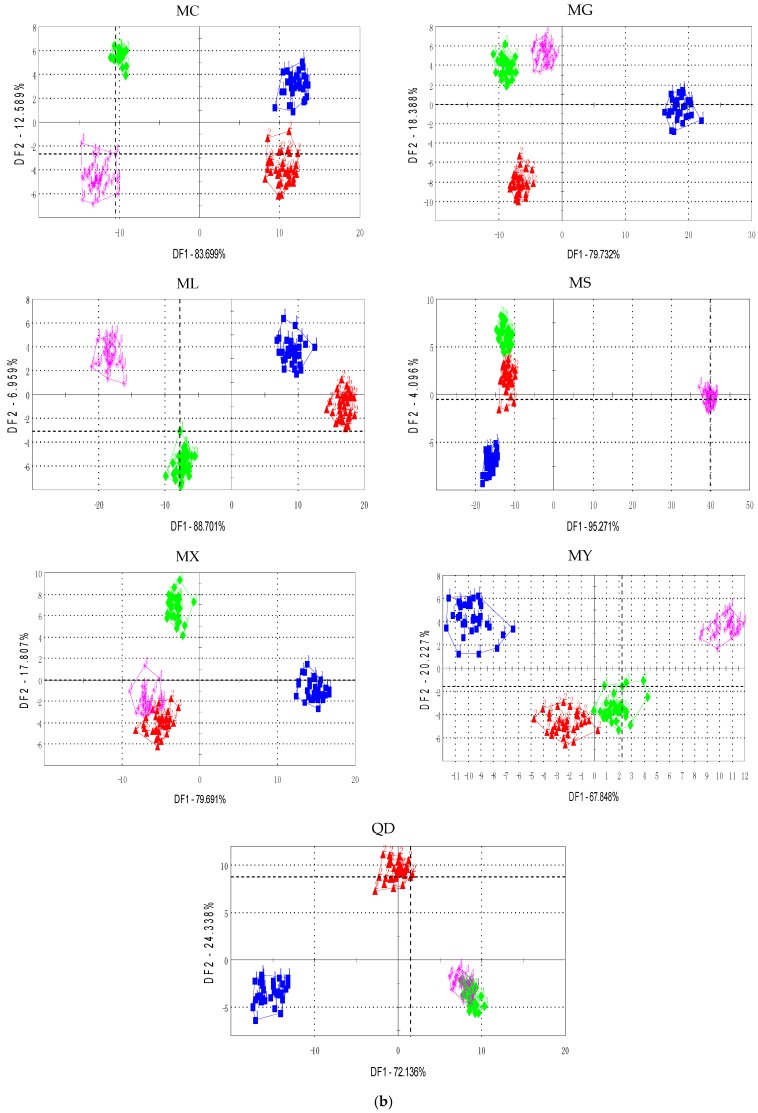
Score plot of the E-nose (**a**) maximum value (MV) (**b**) fused feature.

The discriminant ability of the features was evaluated by cluster scatters performed by linear discriminant analysis. The cluster trends of the tea samples were visualized by a 2-D scatter plot. Figure 2a shows the cluster scatter of the seven tea sample providers by single features (maximum values which were most commonly used) for the electronic nose, and Figure 2b shows the cluster scatter by the fused features. Scatters for the average value, maximum energy, and average energy of the E-nose are provided in the supplementary material.

The results of the electronic nose demonstrated that the tea samples with four grades were not correctly discriminated by a single feature. The boundaries of the classes were not explicit and the samples with different tea grades overlapped. Moreover, the samples in the same class were diverse (i.e., located over a large area). This may decrease the probability of correctly assigning a certain test sample to one of the four grades. In Figure 2b, the scatter plots depict that the fused features significantly improved the performances. It could be seen that for most of the tea samples (i.e., producers of MC, MG, ML, MS, MX, and MY), the tea samples with the same class were much more compact, and the tea samples with different classes were clearly distinguished. Although a number of tea samples from tea producer QD with grade E and grade S overlapped, the samples within the same class became much closer than the scatters of a single feature. The results meet the objective of the dimensionality reduction methods which is to maximize the between-class scatter and minimize the within-class scatter.

**Figure 3:**
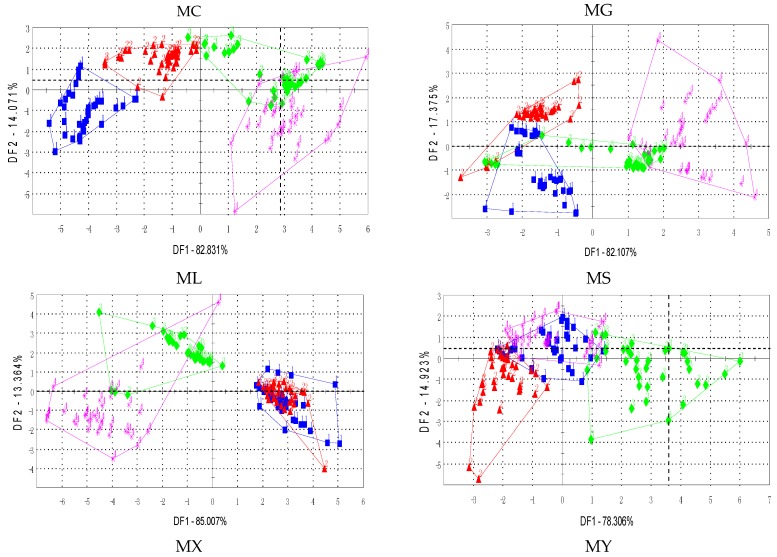

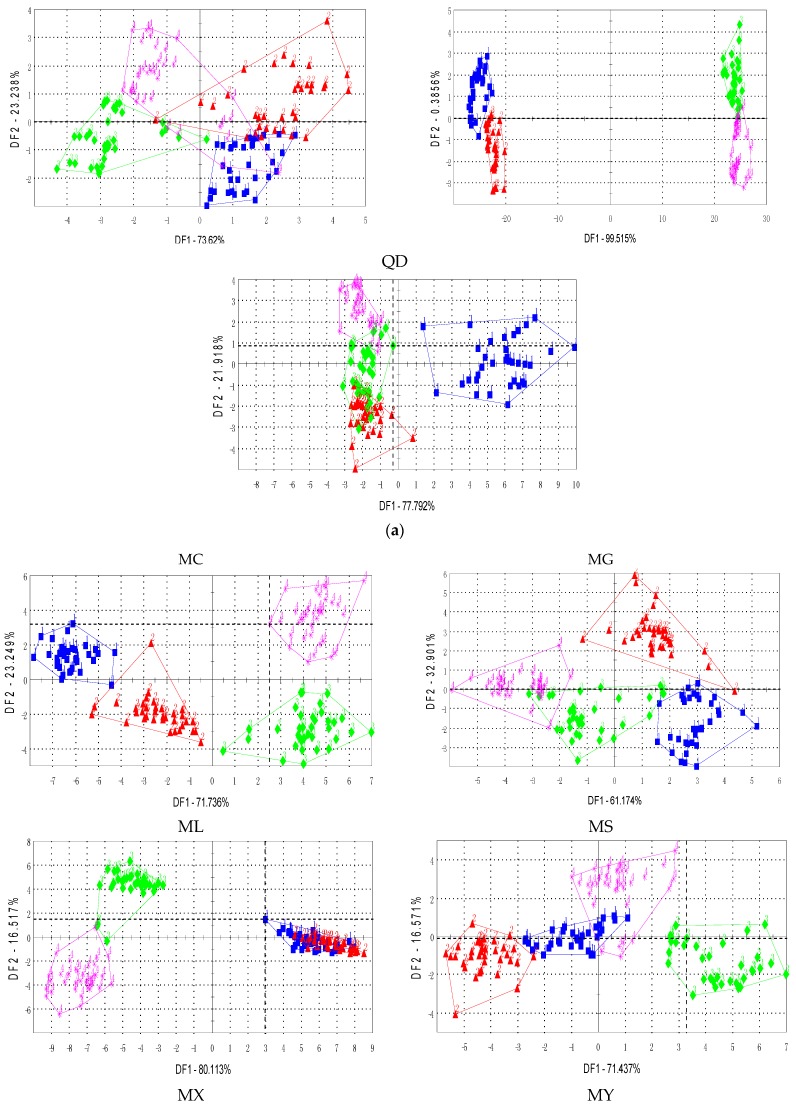

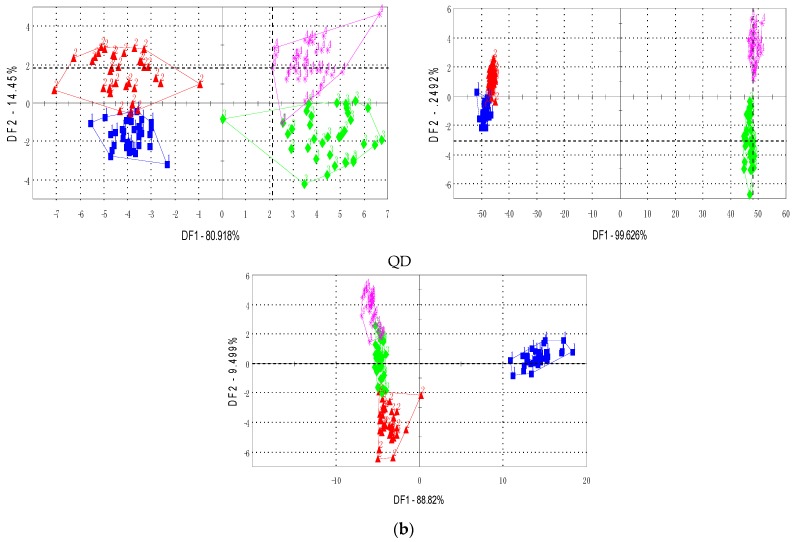
Score plot of the E-tongue (**a**) average value (AV) (**b**) fused feature.

Moreover, Figure 3a illustrated the cluster scatter of the seven tea sample providers by the average values which were commonly used for the electronic tongue, and Figure 3b illustrated the cluster scatter by the fused features.

According to the results of the electronic tongue, the tea samples of the four grades significantly overlapped for single features. The fused feature improved the performance, as shown in Figure 3b. Most of the four grades of tea samples were separated correctly, and few of them were close to the class boundaries. The scatter plots of Figure 3a show that the tea samples from producers MG and MS overlapped for more than two classes, while the fused feature separated the samples clearly and the class boundaries were explicit for each class. The tea samples from producers ML and MX were a mass for a single AV feature, and Figure 3b illustrates that the classes were separated more clearly. Moreover, the samples were closely located within the same class for all the cases.

The results of both the E-nose and E-tongue demonstrate the superiority of the time-domain based and frequency-domain based feature fusion in representing comprehensive signal characteristics. The fused feature could represent the overall characteristics of the sensor signals, and it is more suitable for tea grade identification. However, the results are not clear when putting all of the tea samples from diverse companies together, even in the case of feature fusion. The tea samples collected from this study are much more than state-of-art, and the tea providers are various too. This is the real problem faced in tea gradation in China. To better identify the green tea grades with a large nubmer of tea samples with diverse sources, advanced technologies should be introduced, and that is the purpose of proposing this study.

### 3.2. Dimensionality Reduction of Fused Feature

In this section, the experimental results of the classification performed by feature fusion, the KLDA-based dimensionality reduction, and the KNN classifier were illuminated.

**Figure 4:**
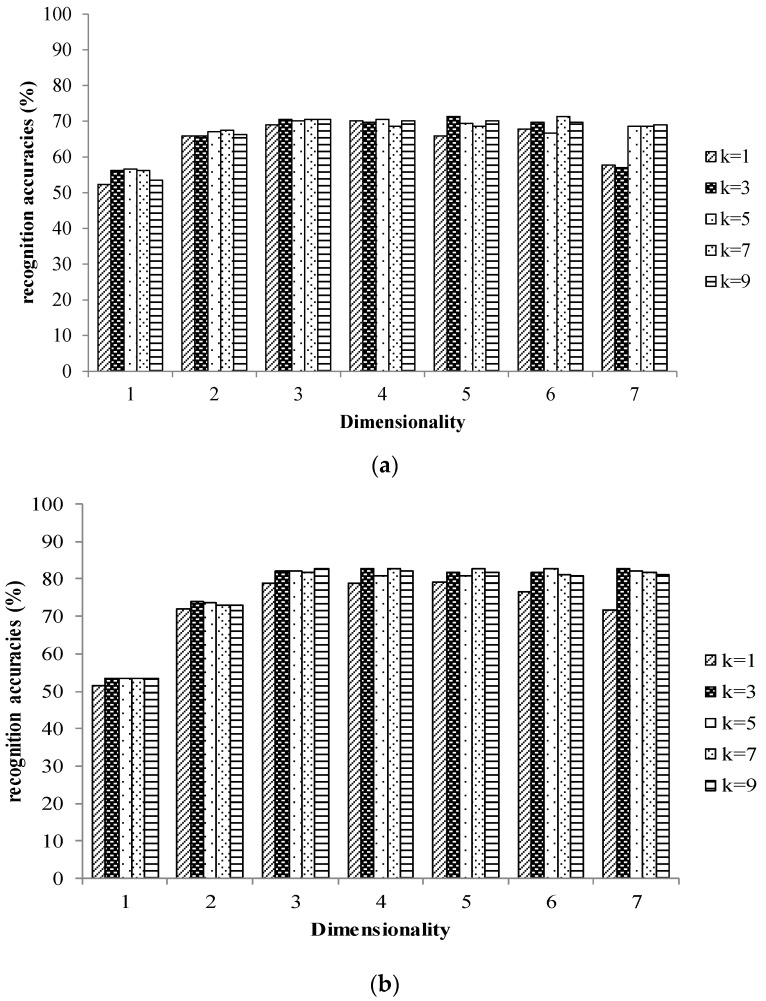
Recognition accuracies of the KLDA-KNN model for (**a**) E-nose (**b**) E-tongue.

The tea samples were divided into two subsets, the training set was utilized to develop the model and the testing set was utilized to verify the performance of the model. The training set consisted of twenty samples of each grade from each tea provider (20×4×7=560 in total) by random selection, and the testing set was constructed from the remaining tea samples. The recognition accuracies versus the dimensionality according to different K values (parameter of the KNN classifier) are shown in Figure 4 for the E-nose and E-tongue, respectively. The recognition accuracies variable versus the reduced dimensions and for different K values. The top recognition accuracy of the E-nose was 71.3%, and it was 82.7% for the E-tongue. The top recognition rate of the E-nose was obtained with Dim=5 and K=3, while that of the E-tongue was obtained with Dim=4 and K=3.

Furthermore, the corresponding confusion matrices of the E-nose and E-tongue are shown in Table 1. Each column of the matrix represents the instances in a predicted class, while each row represents the instance in an actual class. It showed that the tea samples were confused among classes differently. A large number of grade E tea samples were misclassified as grade Y, and many tea samples of grade S were misclassified as grade Y and grand E for the E-nose. While many tea samples of grade T were misclassified as grade Y, grade E samples were misclassified as grade Y, and a great number of tea samples of grade S were misclassified as grade E for the E-tongue.

### 3.3. Decision Level Fusion for Tea Quality Identification

The feature-level fusion, together with the kernel dimensional reduction method, could correctly identify most of the tea samples. However, it was not ideal for all the tea samples collected from the various companies. The D-S evidence-based decision fusion was conducted to improve the classification. As we discussed in Section 2.4.5, the basic probability assignment function was defined by the membership degree from the K-nearest neighbors. In this study, the value K related to the highest recognition accuracy was selected, where the parameter K was set to three for the KNN classifier and the subsequent analysis.

The D-S evidence was conducted on the classification results obtained by feature fusion + KLDA + KNN for both the E-nose and E-tongue. The maximum membership rule defined in Equation (8) was utilized to identify the grade of the tea samples. Based on the multi-level fusion method, the average recognition accuracy achieved for the testing set was 91.3%, compared to 71.3% for the E-nose, and 82.7% for the E-tongue. The corresponding confusion matrix is shown in Table 1. The diagonal of the confusion matrix denoted the correct recognition rates of the four grades, and the other elements denoted the misclassified rates among the four grades. Some tea samples with grade T were misclassified as grade Y (8.0%) for the E-nose, and it was even worse for the E-tongue (16.0%), where the misclassification accuracy reduced to 2.7% for the decision fusion scheme. For the E-nose, the samples of grade Y were misclassified to grade T (19.0%), and for the E-tongue, the misclassification rate was 8.3%. This was improved by the fusion method, producing a value of 1.2%. Moreover, the recognition accuracy of the grade Y samples increased to 94% for the fusion output, and there was no sample misclassified to grade S. The tea samples of grade E were easily misclassified to the other three categories. The recognition accuracy of decision fusion for grade E was enhanced compared to the E-nose and E-tongue. For the E-nose, the number of grade S samples that were misclassified as grade Y and grade E were 18.3% and 20.7%, respsectively. The number of grade S samples that were misclassified as grade Y and E were 4.9% and 8.5% for the E-tongue, respectively. For the fusion method, the grade S tea samples were well identified with a recognition accuracy of 91.5%, while no misclassification occurred for grade T and grade Y samples. It seemed that the multi-level fusion strategy combining the E-nose and E-tongue data significantly improved the recognition accuracy. The grade T, Y, and S samples could be distinguished effectively, and the recognition accuracy for these three grades was above 90% for the large quantity of tea samples.

## 4. Conclusions

A multi-level fusion strategy which combines an electronic nose and electronic tongue was proposed and applied to tea quality identification. The procedure included feature-level fusion (fuse the time-domain based feature and frequency-domain based feature) and decision-level fusion (D-S evidence to combine the classification results from KNN). The experiments were carried out on tea samples collected from various tea providers with four grades which made it very difficult, and the results showed a much better classification ability for the multi-level fusion system. The proposed algorithm could better represent the overall characteristics of the tea samples for both odor and taste, and that was the reason why the system outperformed the single feature method or single classifier.

Furthermore, the general idea of this paper can be improved by selecting better feature representing methods, and more attributes like appearance may be considered for this multi-level fusion system. This paper provides a framework for the combination of features and decisions, and it is important consider the difference sources to better identify the tea quality.

## Figures and Tables

**Table 1:** Confusion matrix (%) for KLDA-KNN classification of the E-nose, E-tongue, and decision fusion.

	E-Nose Feature				E-Tongue Feature				Decision Fusion			
	T	Y	E	S	T	Y	E	S	T	Y	E	S
T	92.0	8.0	0	0	78.7	16.0	5.3	0	93.3	2.7	4.0	0
Y	19.0	78.6	1.2	1.2	8.3	85.7	4.8	1.2	1.2	94.0	4.8	0
E	2.5	26.6	58.2	12.7	1.3	8.9	82.3	7.6	1.3	3.8	86.1	8.9
S	4.9	18.3	20.7	56.1	2.4	4.9	8.5	84.1	0	0	8.5	91.5

## Supplementary Materials

The supplementary materials are available online at http://www.mdpi.com/1424-8220/17/5/1007/s1.
