# Supplementary material for: A Framework for the Multi-Level Fusion of Electronic Nose and Electronic Tongue for Tea Quality Assessment
Source: Sensors (Basel). 2017 May 3;17(5):1007. doi: 10.3390/s17051007 (PMC5469530; doi:10.3390/s17051007)

Supplementary Material

Figure #1. Score plot by the average value (AV) of E-nose.

MC

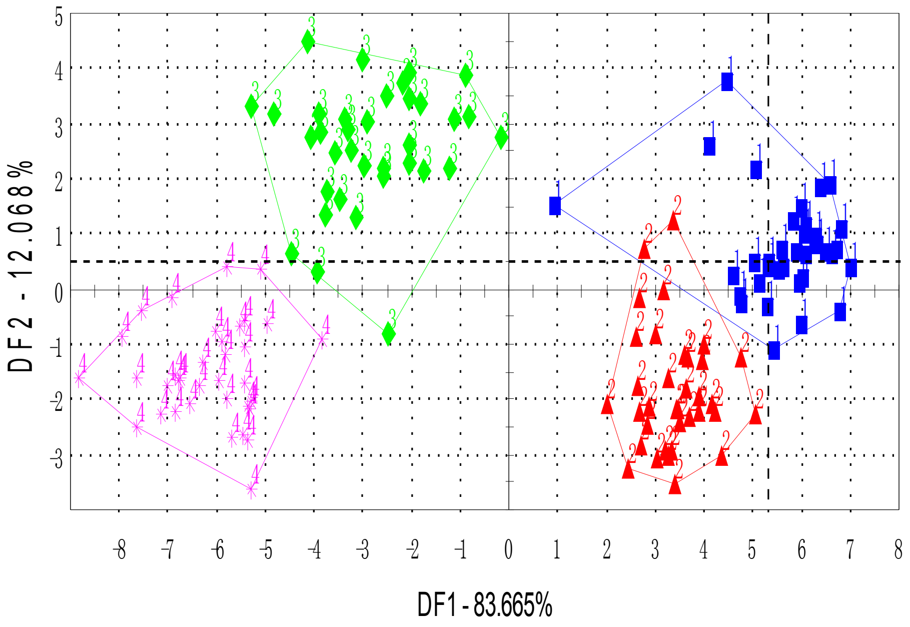

MG

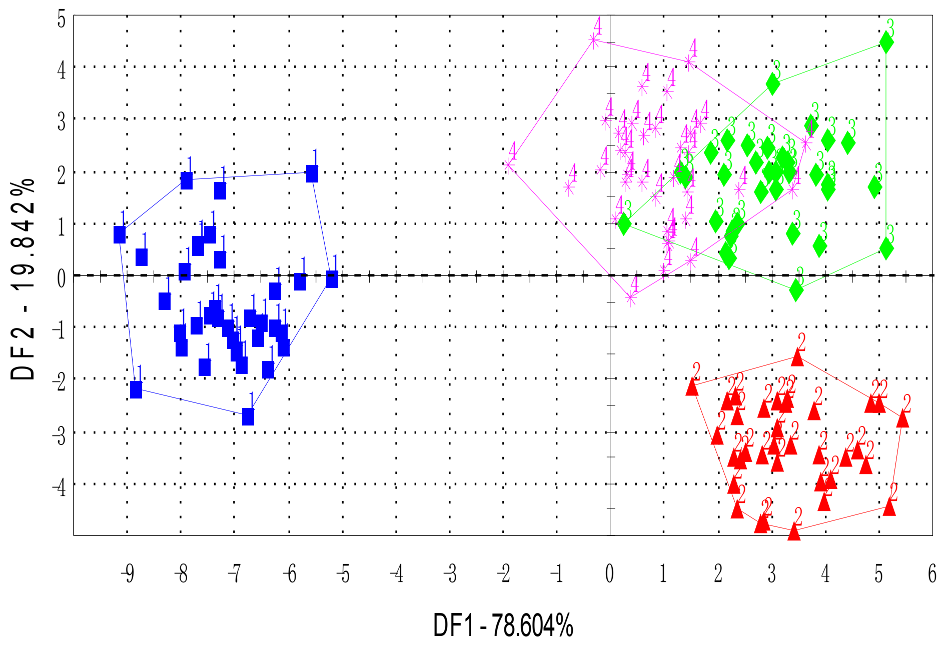

ML

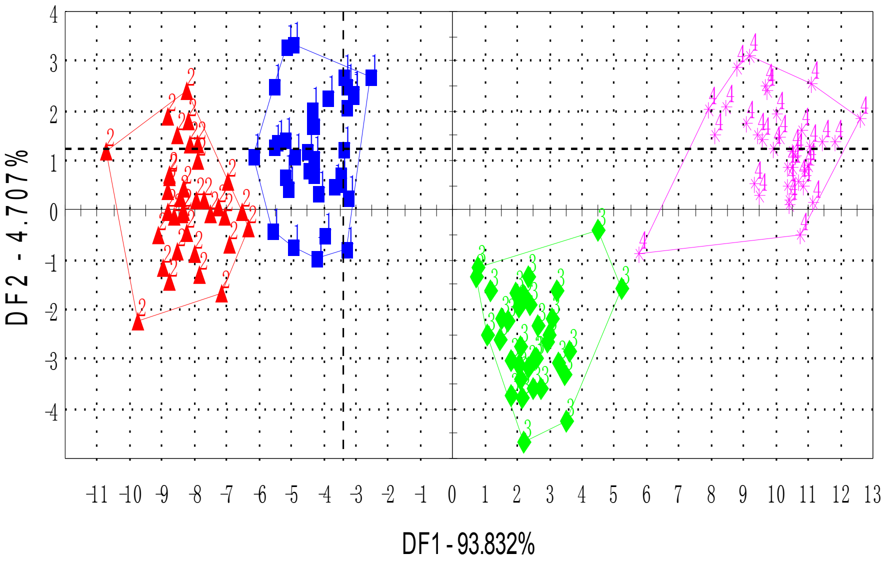

MS

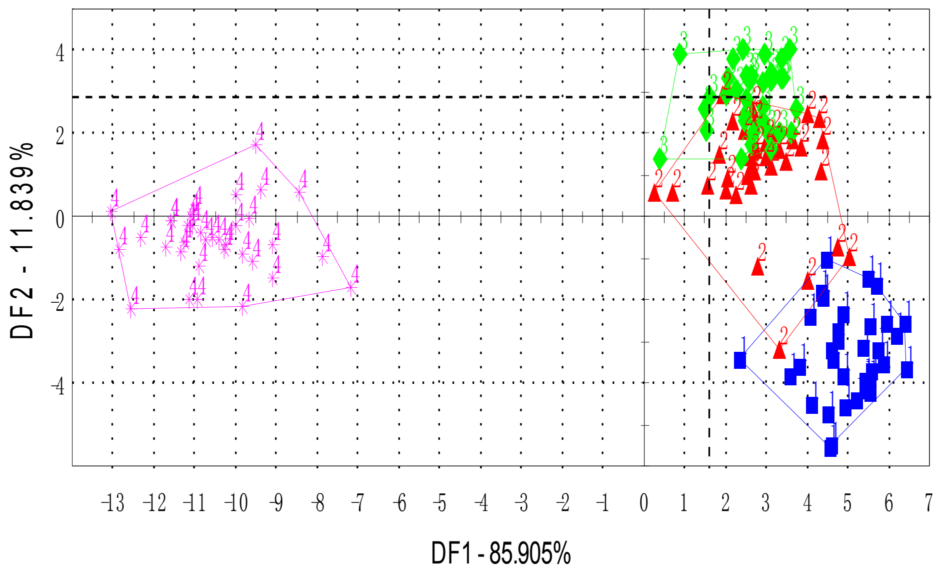

MX

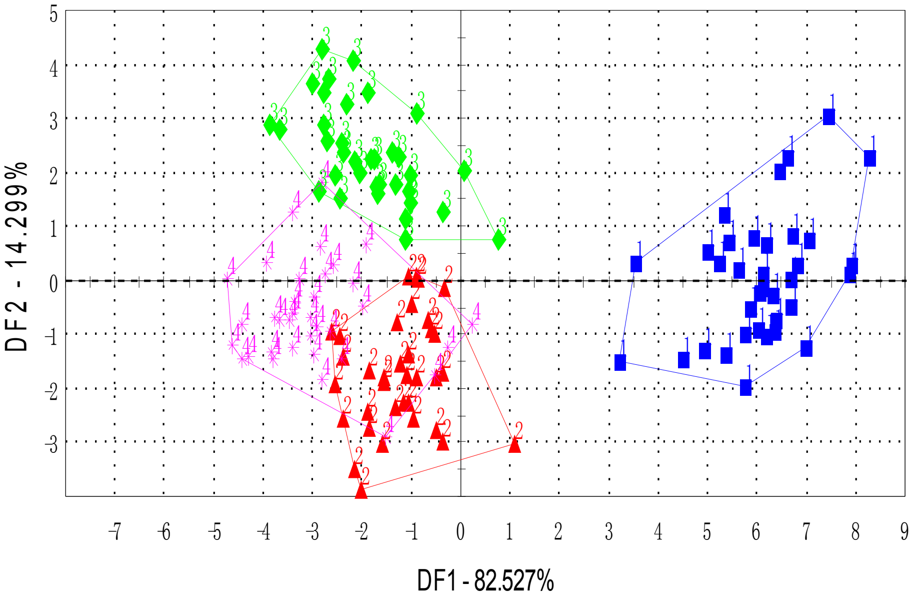

MY

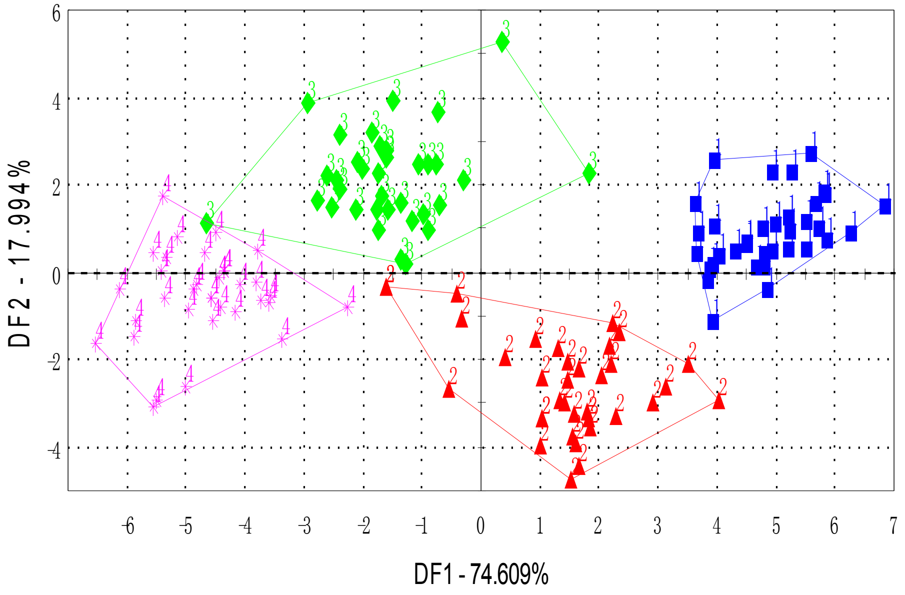

QD

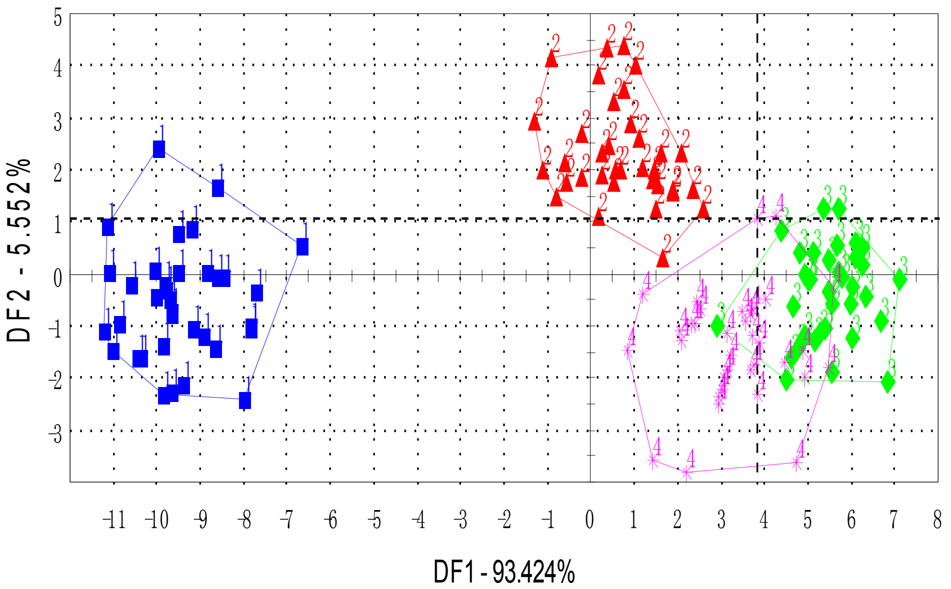

Figure #2. Score plot by the maximum energy (ME) of E-nose.

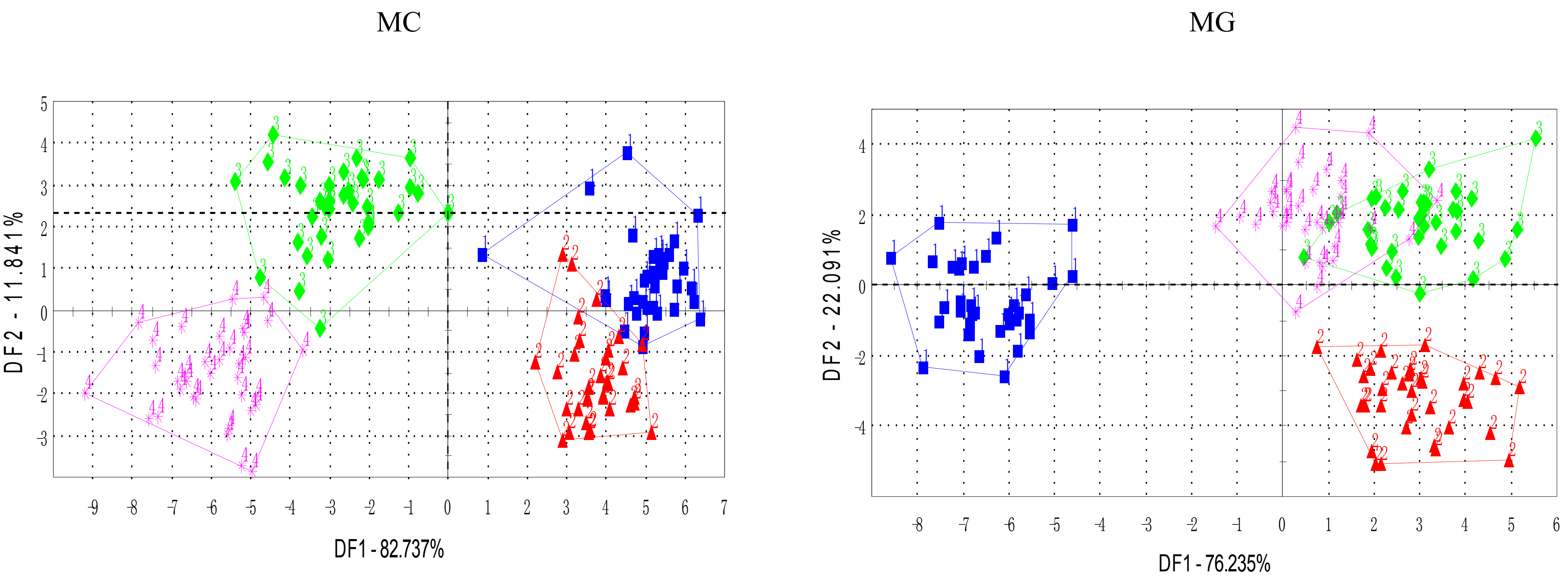

ML

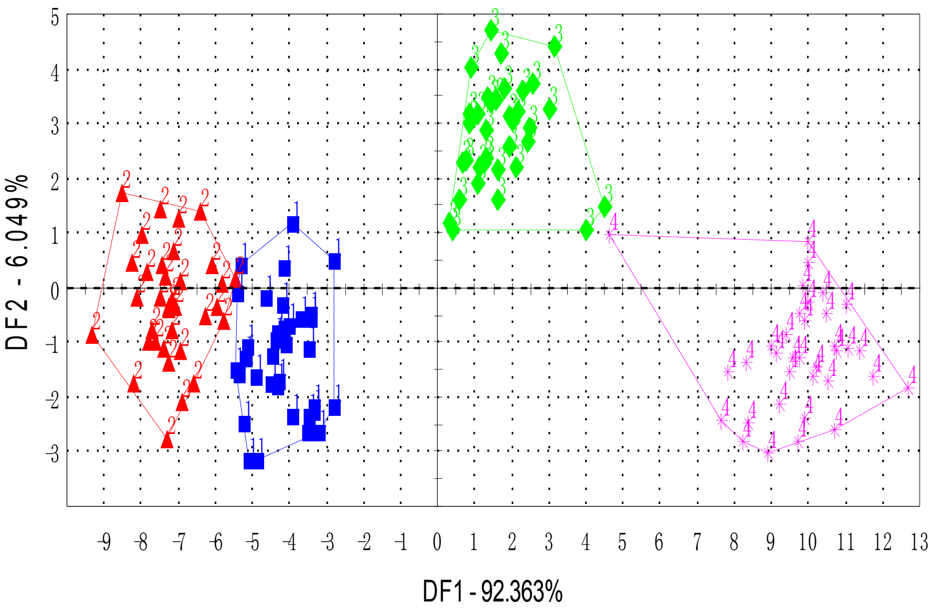

MS

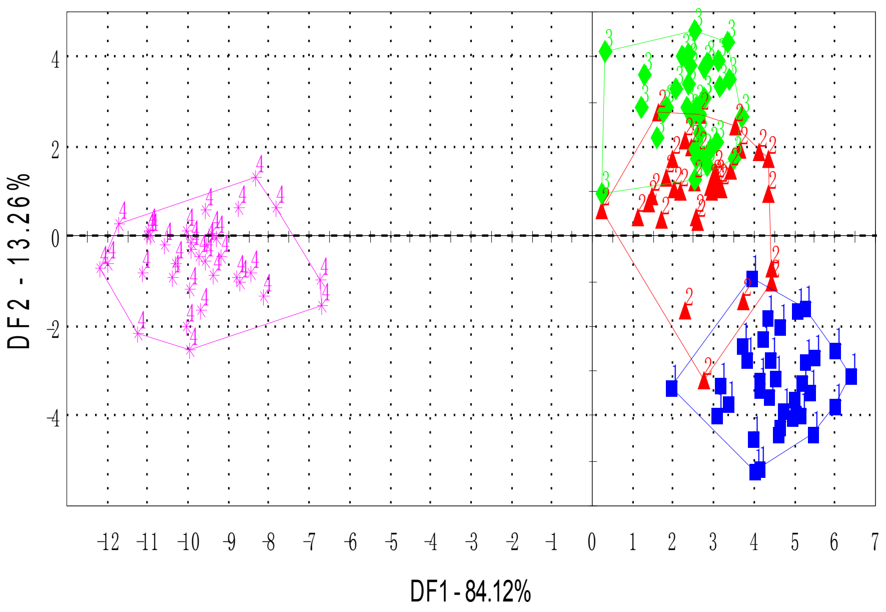

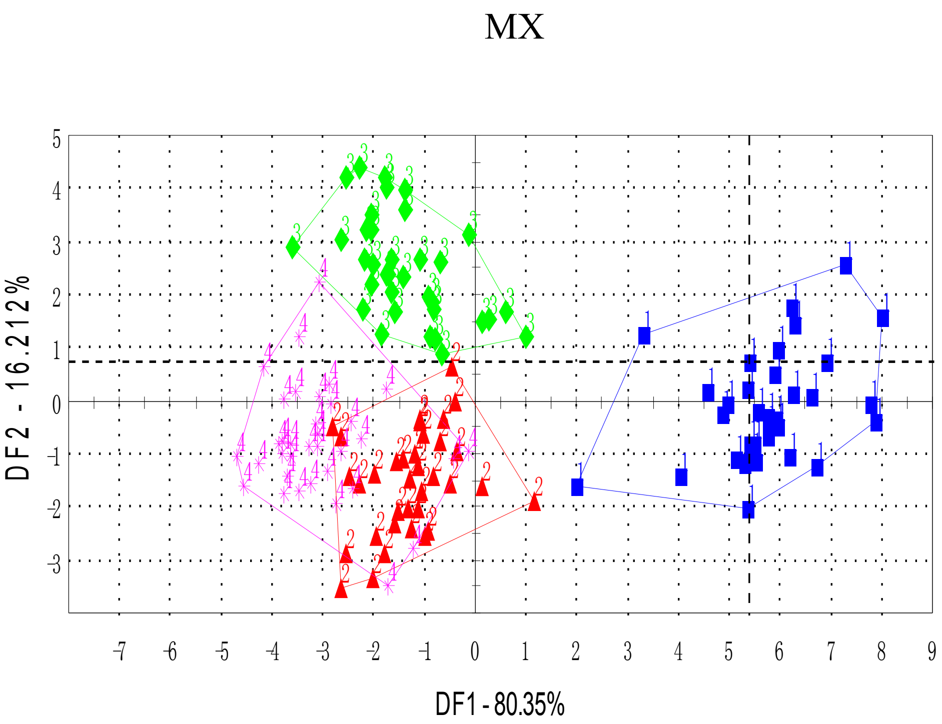
MX
DF 2 - 16.212%
DF1 - 80.35%

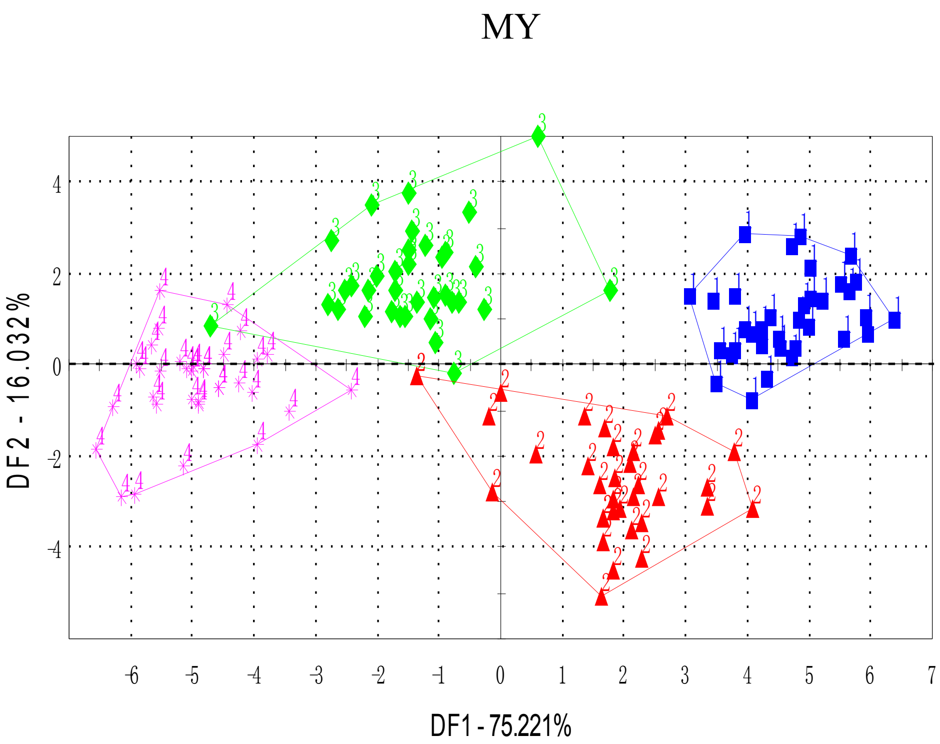
MY
DF 2 - 16.032%
DF1 - 75.221%

QD

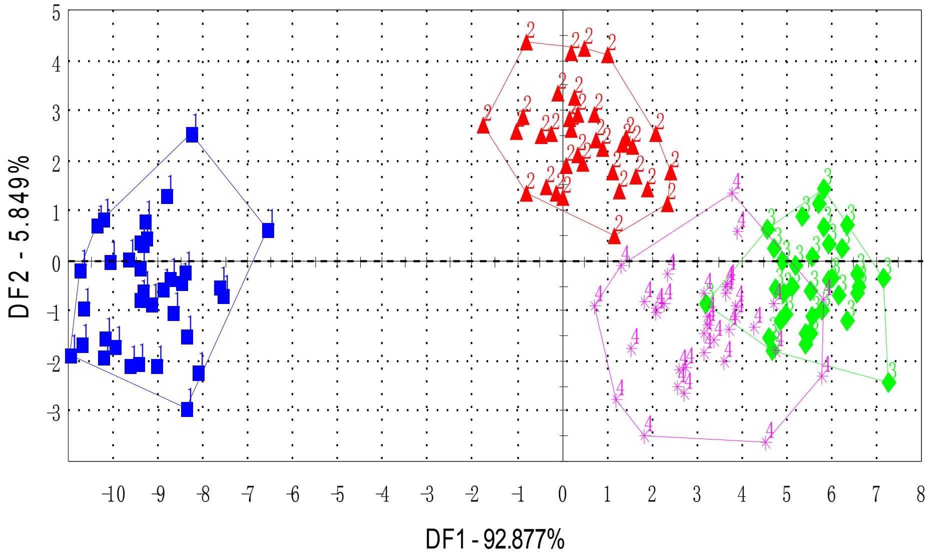

Figure #3. Score plot by the average energy (AE) of E-nose.

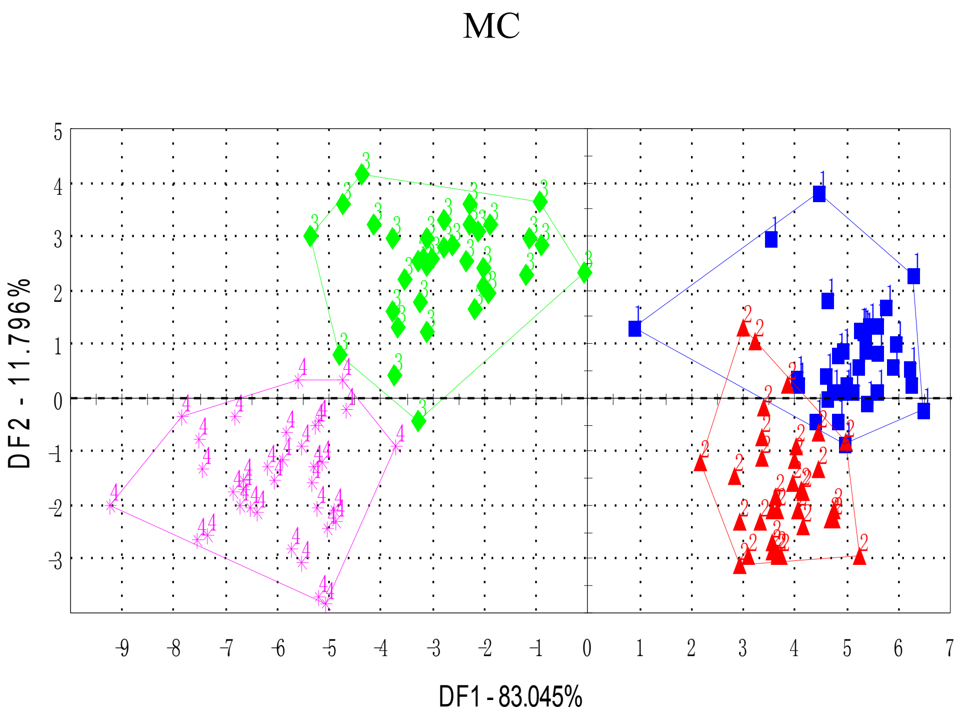

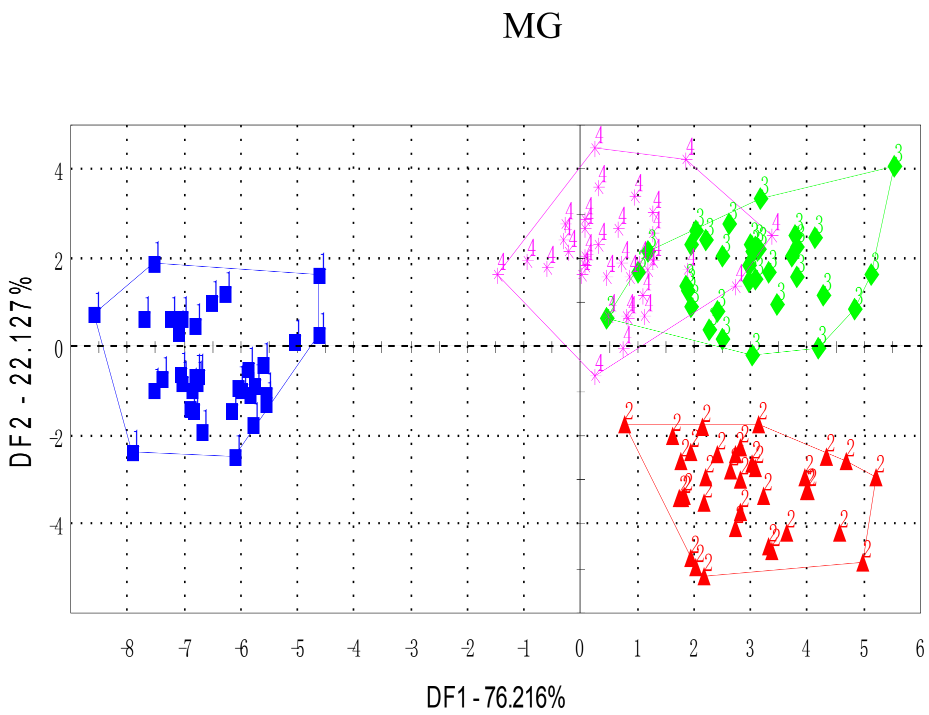

ML

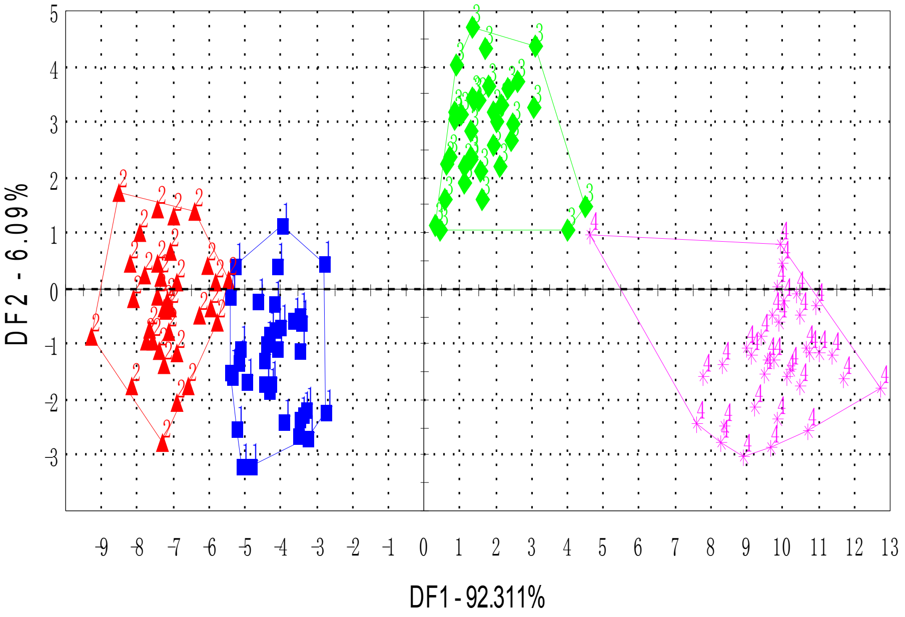

MS

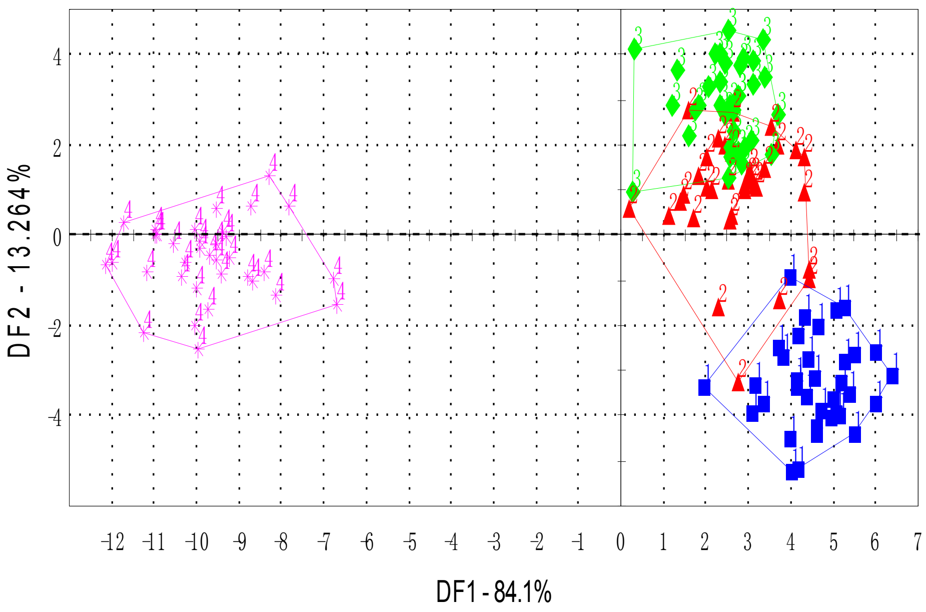

MX

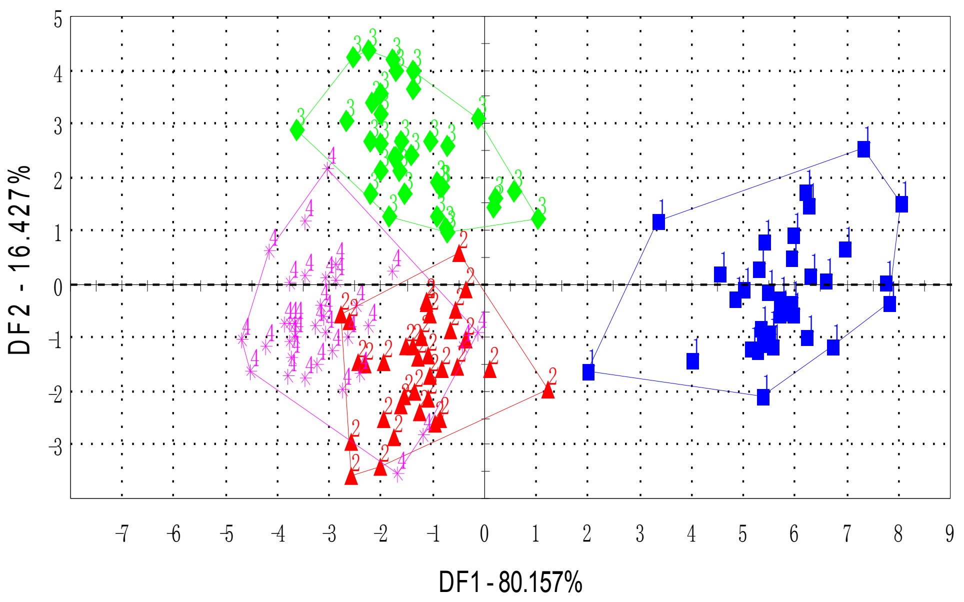

MY

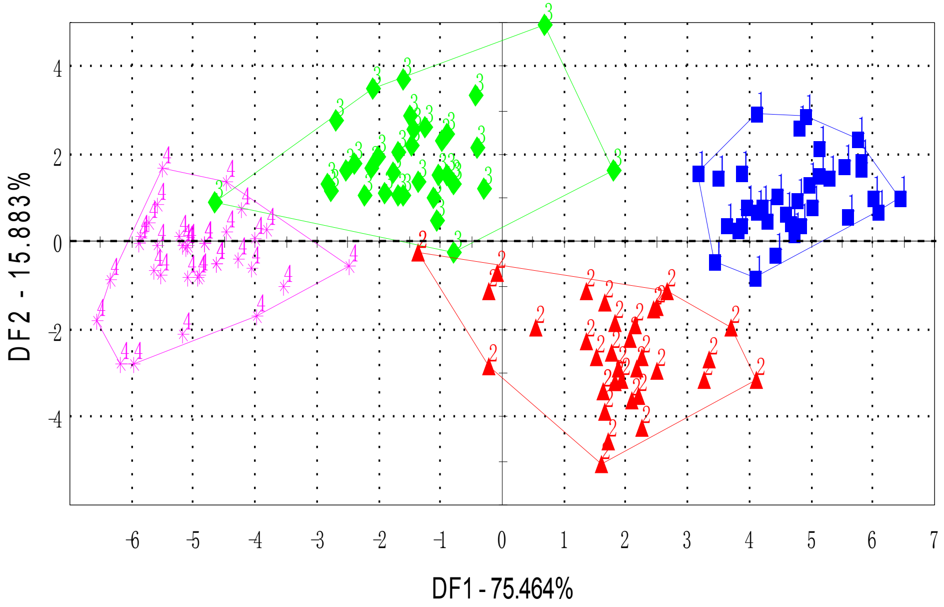

QD

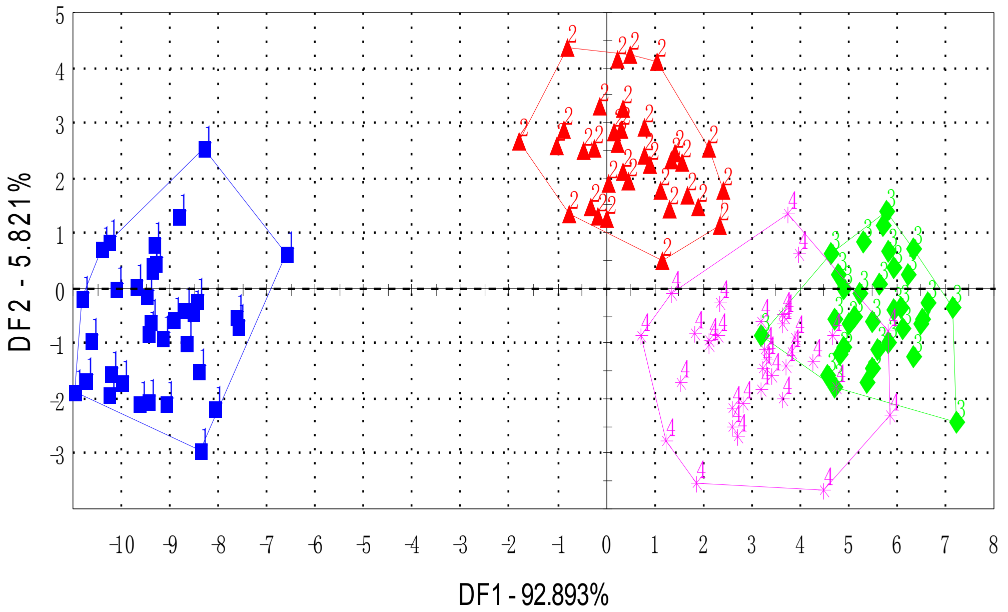

Figure #4. Score plot by the maximum value (MV) of E-tongue.

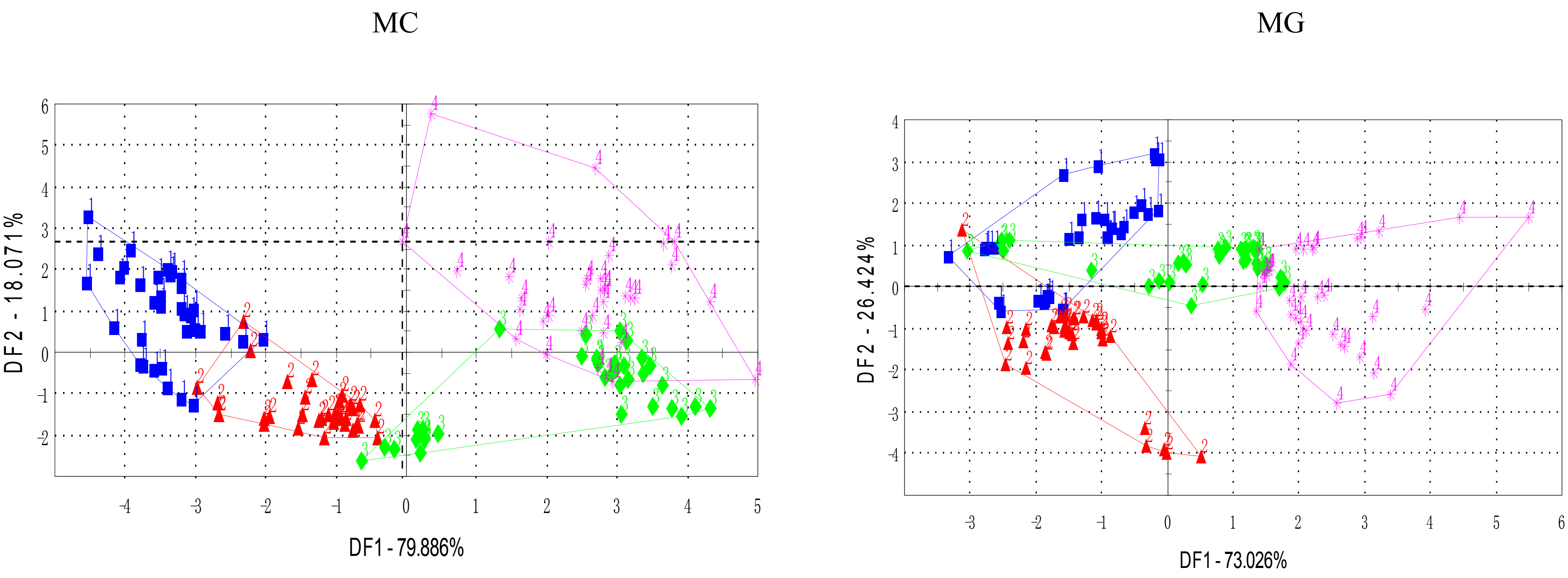

ML

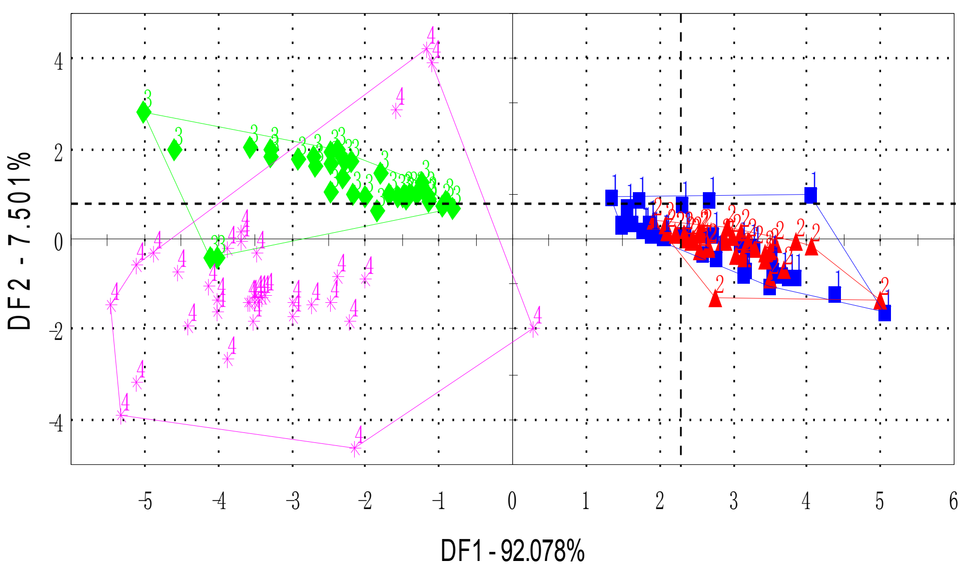
DF2 - 7.501%
DF1 - 92.078%
4
2
0
-2
-4
-5 -4 -3 -2 -1 0 1 2 3 4 5 6

MS

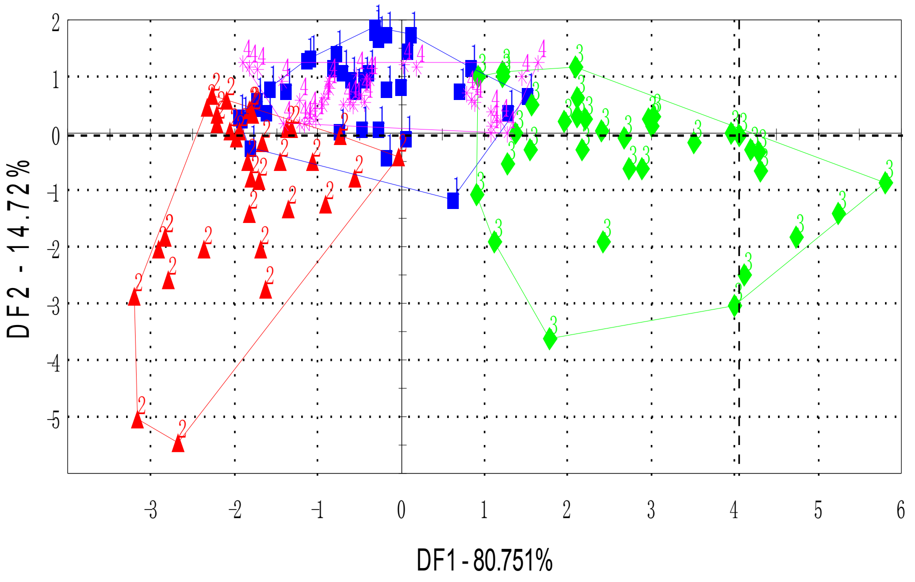
DF2 - 14.72%
DF1 - 80.751%
2
1
0
-1
-2
-3
-4
-5
-3 -2 -1 0 1 2 3 4 5 6

MX

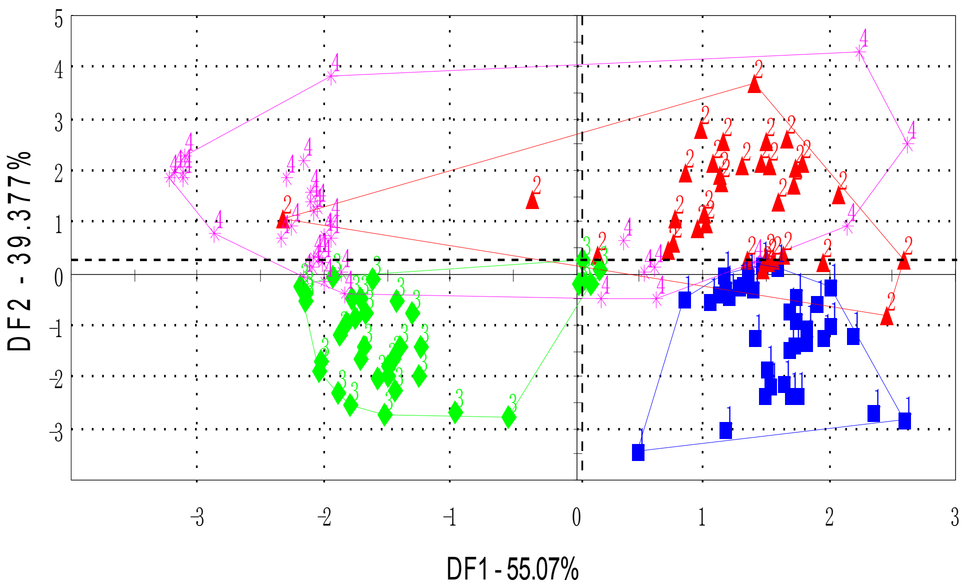

MY

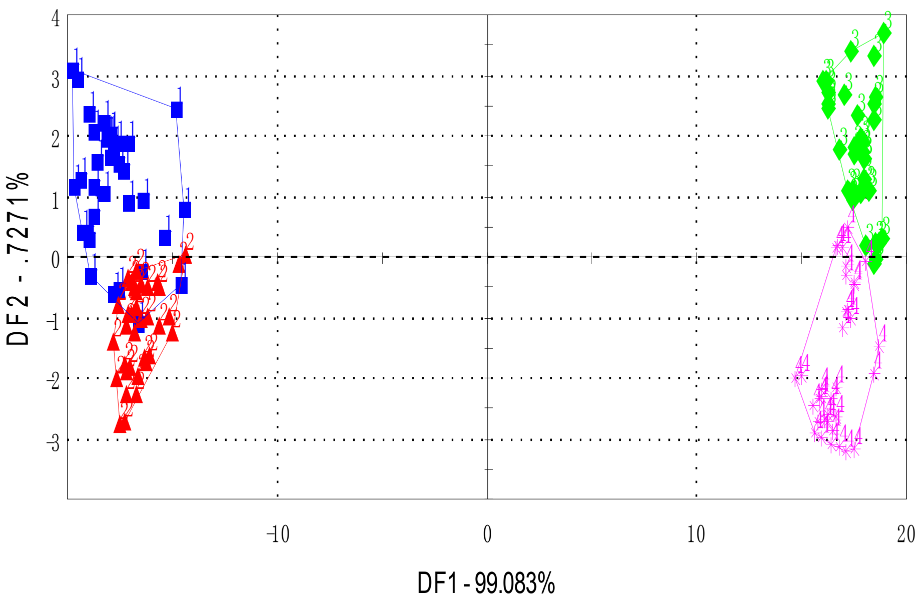

QD

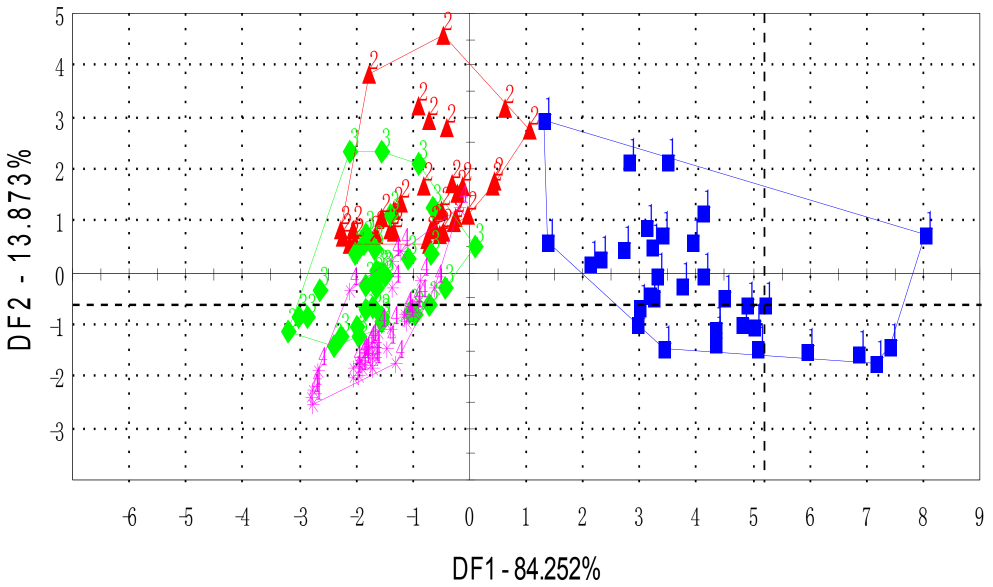

Figure #5. Score plot by the maximum energy (ME) of E-tongue.

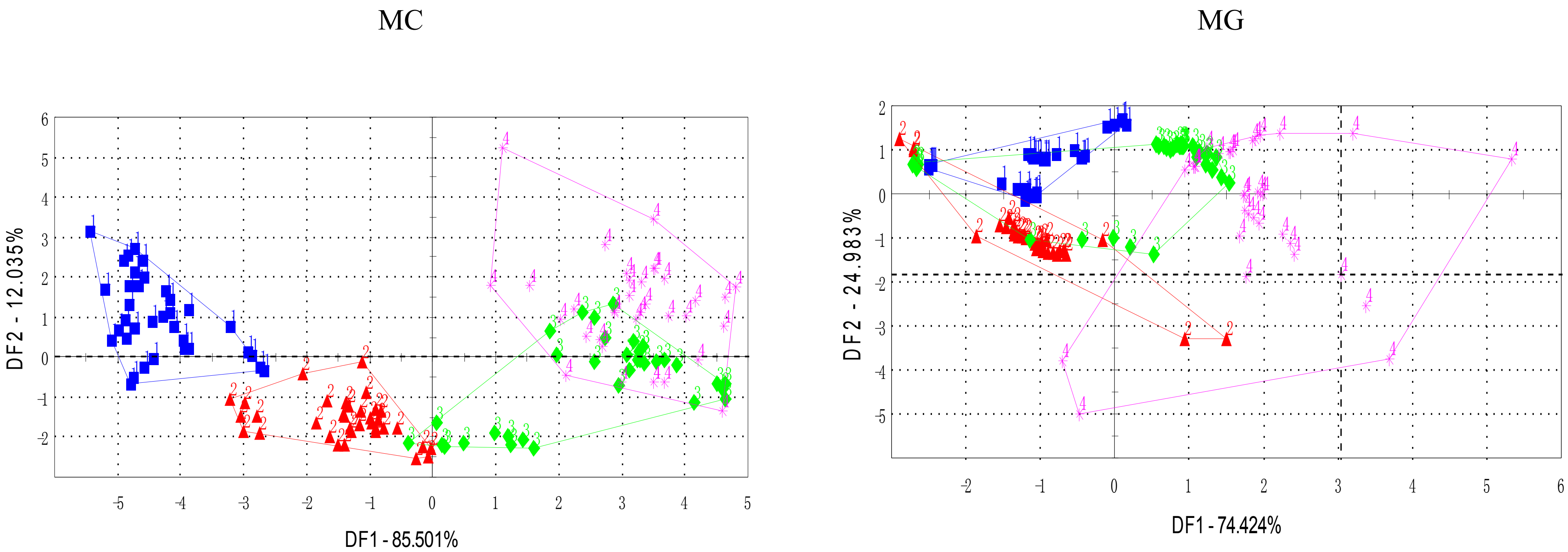

ML

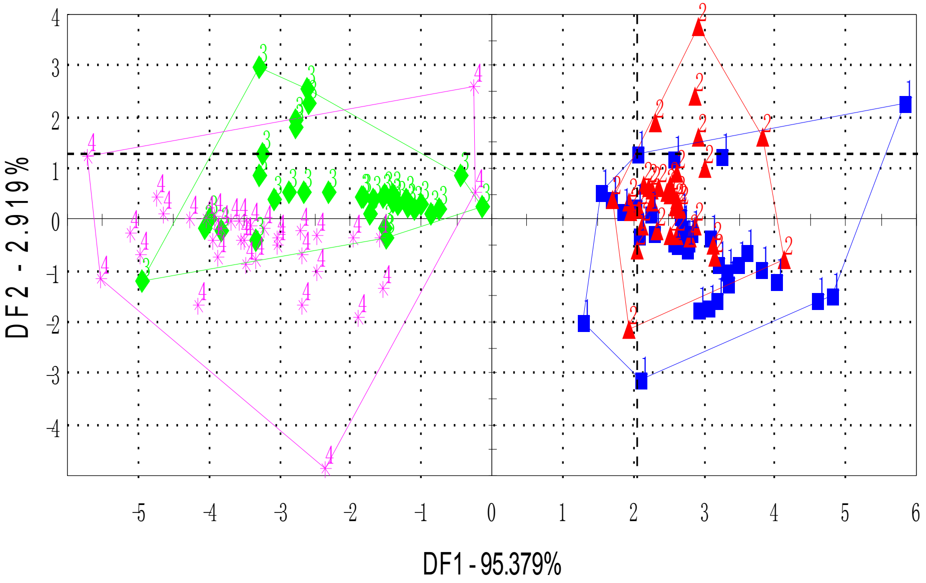

MS

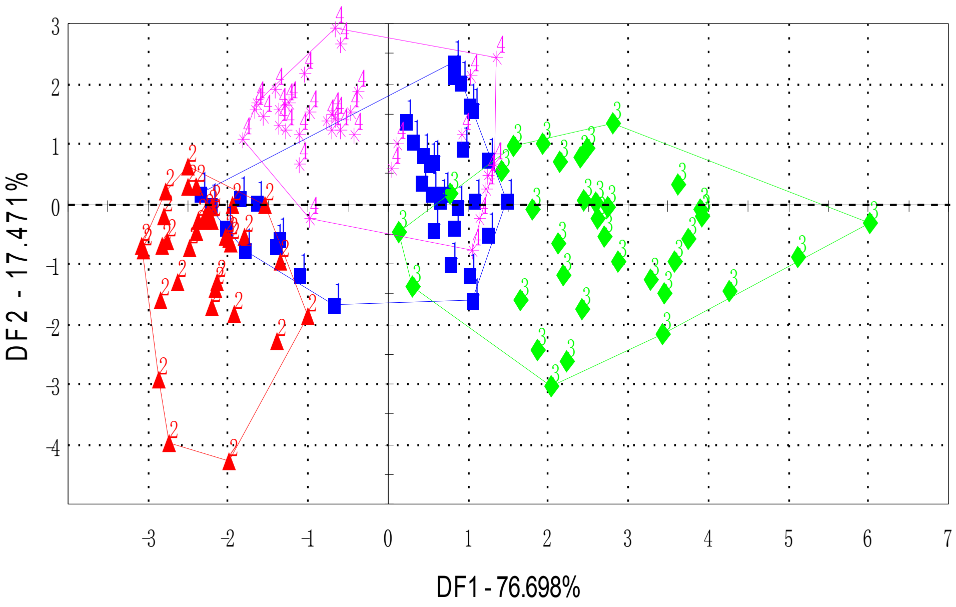

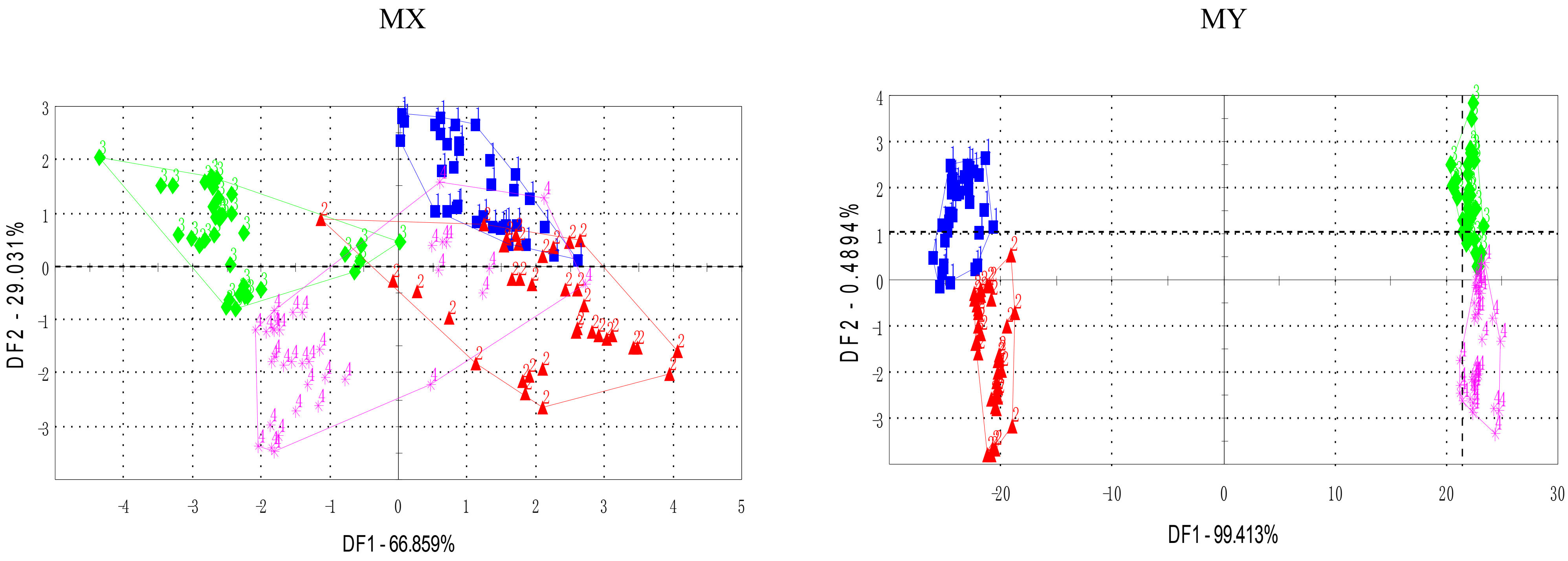

MX
DF2 - 29.031%
DF1 - 66.859%
MY
DF2 - 0.4894%
DF1 - 99.413%

QD

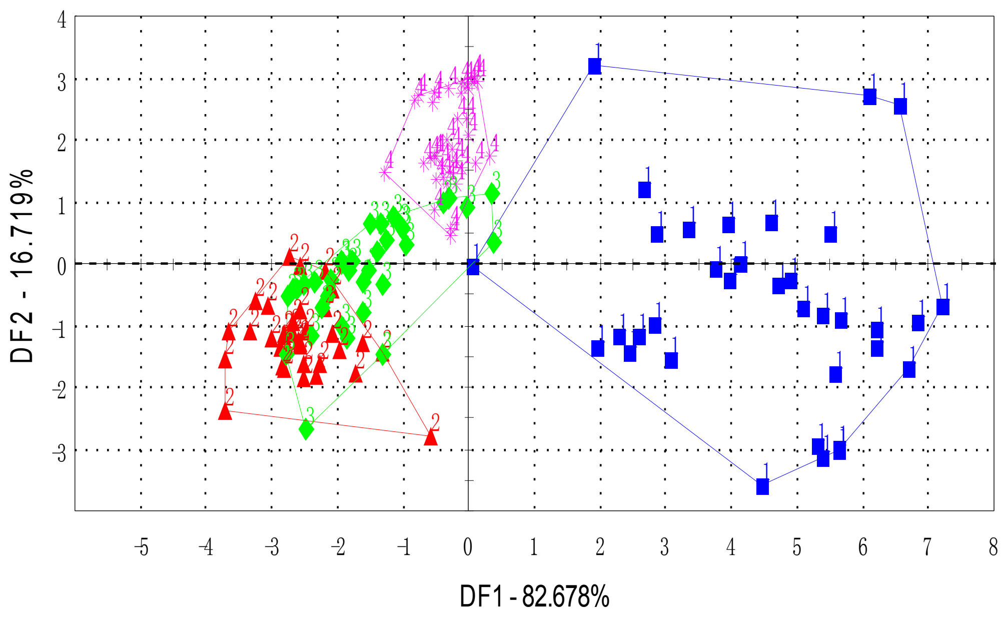

Figure #6. Score plot by the average energy (AE) of E-tongue.

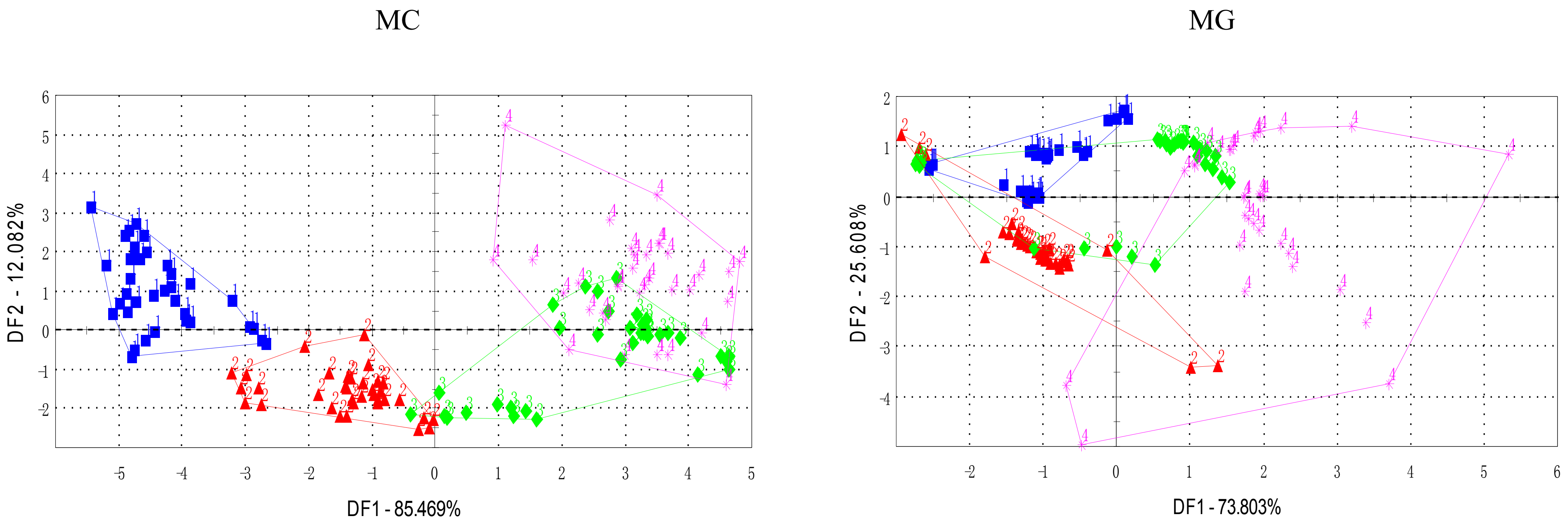

ML

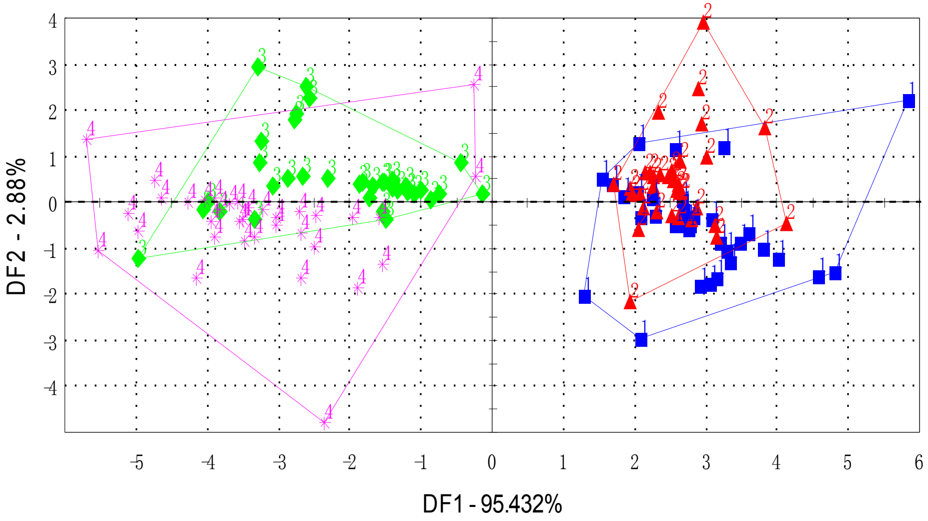

MS

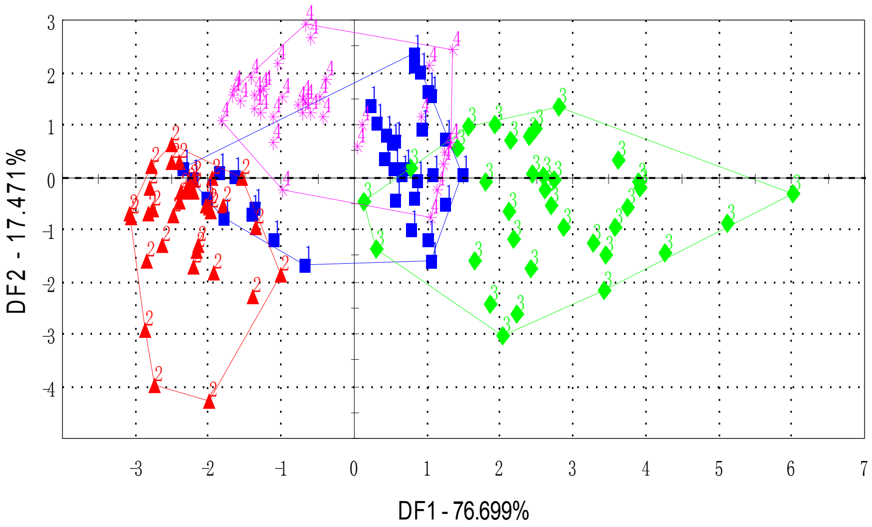

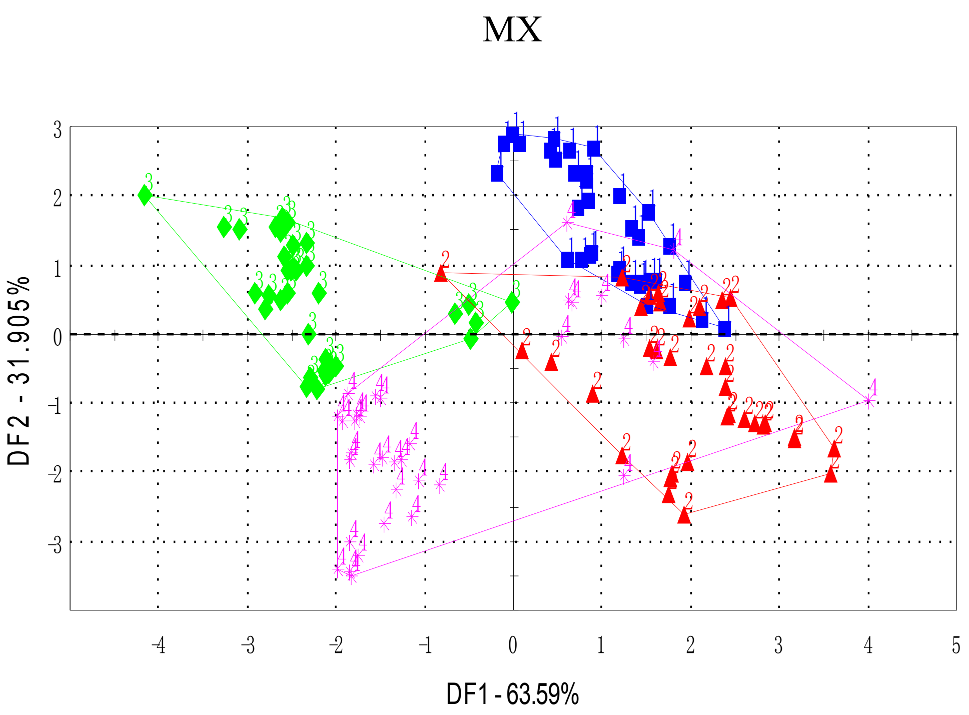
MX
DF2 - 31.905%
DF1 - 63.59%

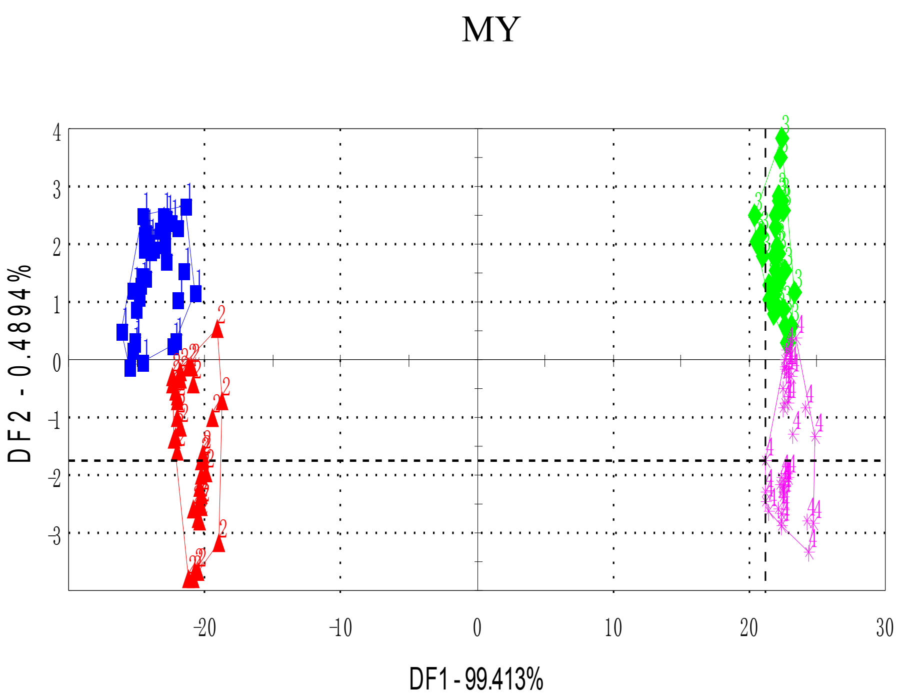
MY
DF2 - 0.4894%
DF1 - 99.413%

QD

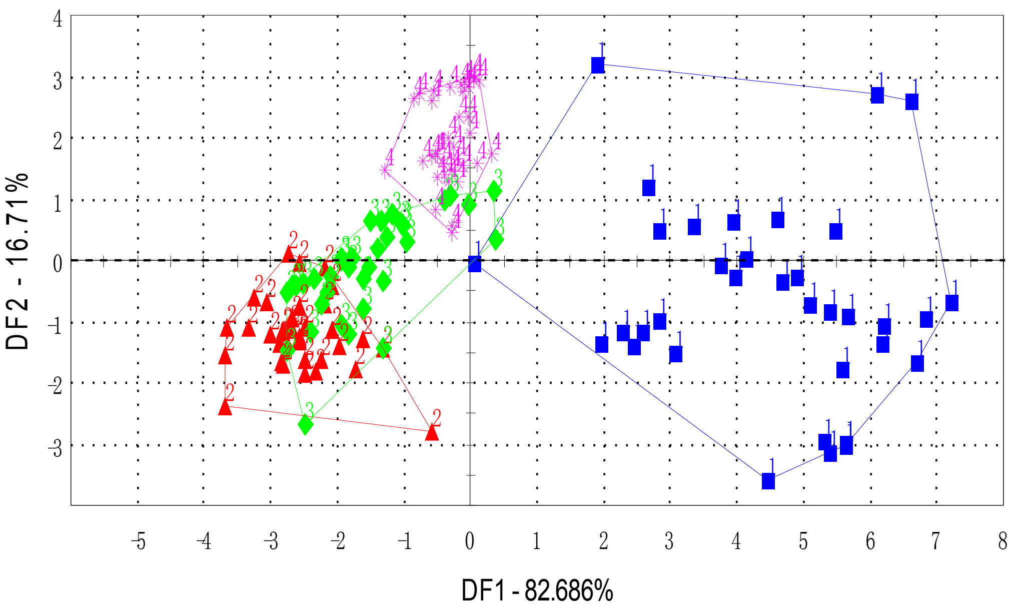

Supplement: Supplementary file 1 [file sensors-17-01007-s001.pdf]
